# A Versatile Single-Step Micro- to Nanoparticles Laser Graphitization-Driven Conversion Route for Graphene-Embedded Nanoparticle Composites

**DOI:** 10.1021/acsami.6c00908

**Published:** 2026-03-11

**Authors:** Assaf Eran, Gil Daffan, Fernando Patolsky

**Affiliations:** † Department of Materials Science and Engineering, Faculty of Engineering, 26745 Tel Aviv University, Tel Aviv 69978, Israel; ‡ School of Chemistry, Faculty of Exact Sciences, Tel Aviv University, Tel Aviv 69978, Israel; § Tel Aviv University Center for Nanoscience and Nanotechnology, Tel Aviv University, Tel Aviv 69978, Israel

**Keywords:** laser-induced graphene, microparticles, silicon, nanocomposites, lithium-ion batteries, anodes

## Abstract

Traditional top-down nanoparticle synthesis is often limited by the high energy demands and costs of mechanical milling and high-power pulsed lasers. Beyond synthesis, integrating these powders into functional devices remains a significant challenge, typically requiring cumbersome, multistep procedures to incorporate particles into conductive support matrices. Building on the established foundation of laser-induced graphene (LIG) synthesis, we introduce a versatile single-step methodology utilizing low-power continuous laser irradiation of phenolic resin blended with microparticle precursors (e.g., Si, SiO, and Mg) to produce functional nanocomposites under ambient conditions. We propose that ultrafast laser-induced photothermal graphitization drives an explosive boiling mechanism, where rapid localized heating converts microparticles into nanoparticles. These ejected molten nanoparticles are simultaneously embedded within the as-formed porous LIG scaffold. Validated across diverse precursors, this versatile process results in a monolithic, self-supporting composite with strong interfacial coupling achieved without binders or postprocessing. Notably, a “laser-milled” SiO/LIG anode synthesized from microprecursors demonstrated performance comparable to analogous anodes fabricated using premade nanoparticles, yielding a lithium-ion battery with a reversible capacity exceeding 1400 mAh/g over 105 cycles at C/7 and 79% retention over 350 cycles at 1.28C. This scalable strategy represents the first investigation into converting raw micropowders and commercial polymers into functional nanoparticle–graphene composites in a single, streamlined step.

## Graphical abstract

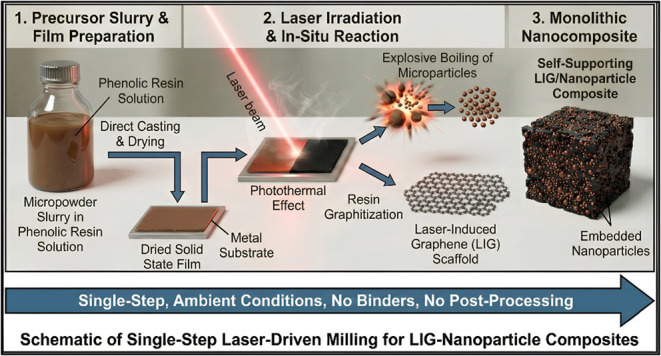

## Introduction

Particles ranging in size from 1 to 100 nm occupy a unique category, positioned on the boundary between single molecules and bulk materials, and are therefore aptly defined as nanoparticles (NPs). Their small scale imparts unique properties or greatly enhances those found in the bulk material.^1^ This effect is largely due to the high surface area-to-volume ratio, which emphasizes surface effects that enhance reactivity and other properties, as well as quantum confinement effects that arise from restrictions on electron mobility in one or more dimensions. Nanoparticles are categorized by their dimensionality: they can be defined as 0D (e.g., quantum dots and nanoparticles), 1D (e.g., nanorods and nanotubes), or 2D (e.g., nanosheets such as graphene) nanomaterials.^2^ These new and enhanced properties make nanoparticles invaluable and, in many cases, irreplaceable, with demand for them steadily increasing.^3^

The possible applications for nanoparticles encompass every field of science and engineering, and most prominent among them are applications in medicine, energy, environmental remediation, and catalysis.^4–6^ In medicine, nanoparticles and other nanostructures can be used in drug delivery,^7,8^ as they can penetrate the intestines or the blood–brain barrier, whereas larger particles of the same type could not. They are also employed in extremely sensitive sensing applications, enabling the miniaturization of medical devices.^9^ In catalysis and environmental applications, nanoparticles are used to vastly increase reaction rates, for example, in chemical reactions or water purification,^10,11^ while simultaneously minimizing the use of expensive catalysts. In energy applications, nanoparticles can improve the kinetics of battery active materials and enable the utilization of materials that are otherwise limited by their bulk properties.

A prime example of the latter is the application of silicon (Si) in lithium-ion batteries. Si is an excellent host for lithium ions as an anode material, capable of storing approximately ten times more charge per unit mass than graphite.^12^ However, Si faces several fundamental challenges that hinder its application in battery technologies. One major challenge is the significant volume expansion (300–400%) Si undergoes during lithiation, which leads to mechanical degradation, loss of electrical contact, and instability of the solid electrolyte interphase (SEI), ultimately causing a rapid capacity fade. Additionally, the low intrinsic electrical conductivity of Si further hampers its performance as an anode. Employing Si in nanostructured forms can alleviate these drawbacks by accommodating volume changes more effectively and improving conductivity at the nanoscale.^13,14^

The benefits of nanomaterials necessitate the development of new synthesis strategies, which currently bottleneck their scalability and commercial viability.^15^ Many established techniques for producing nanoparticle-based composites rely on the dimensionality reduction of commercial micropowder precursors via top-down approaches, such as ball milling. These mechanical techniques reduce microparticles to the nanoscale via sustained, high-energy collisions. Although widely used, ball milling and related techniques are typically energy-intensive, time-consuming, and inherently multistep processes. They also offer limited control over particle size distribution and morphology, while often requiring high-purity precursors to prevent contamination.^16^ In contrast, bottom-up methods, based on the assembly of nanoparticles from molecular or atomic building blocks, can offer more precise control but are often complex, costly, and difficult to scale. Additionally, NP synthesis introduces technical challenges for the production of composite materials. Beyond the inherently higher cost of nanoparticles compared to bulk powders, their low tap density, tendency to agglomerate, increased health risks, and heightened reactivity all necessitate specialized care and handling.^17^

For energy applications, nanoparticles are typically incorporated into electrochemical architectures, where they are strongly embedded in a matrix that provides the required properties, such as electrical conductivity and mechanical stability.^18^ However, embedding nanoparticles in the required matrix presents another challenge for their application. The common approach involves dispersing NPs in a liquid medium (e.g., alcohols and organic compounds like NMP) and incorporating binders along with other functional materials that impart desired properties, for example, carbon black or carbon nanotubes for electrical conductivity. While widely used and scalable, this approach adds multiple steps to the production process, such as formulating stable inks and requiring additional materials (e.g., binders, surfactants, and rheology modifiers). Consequently, new methodologies have been developed to reduce the complexity of the composite electrode fabrication.

In this context, laser-induced graphene (LIG) offers a straightforward and efficient method for producing conductive, porous graphene directly onto various substrates.^19^ This process involves the rapid heating of carbon-rich precursors, such as polyimide or phenolic resin, through ultrafast ambient laser irradiation, leading to localized pyrolysis.^20^ During this pyrolytic process, carbon atoms reorganize into a graphitic structure, while gases, mainly CO, CO_2_, and H_2_, evolve.^21,22^ This gas evolution creates a highly porous architecture, increasing surface area and enhancing conductivity.

Notably, previous works have also explored several approaches for embedding active components into the LIG matrix, in which suitable precursors are first blended with an LIG-compatible carbon source and subsequently transformed during ultrafast photothermal graphitization. Previous studies have highlighted the incorporation of premade Si nanoparticles into the LIG matrix,^23–25^ the in situ molecular dispersion and covalent binding of sulfur or phosphorus adducts within the graphene,^26,27^ and the bottom-up nanoparticle synthesis of metal nanoparticles from metal salts blended with LIG precursors through in situ metal ion reduction.^28^

Unlike traditional graphene synthesis methods that often require high temperatures, chemical treatments, or extensive processing steps, LIG formation is relatively simple. It occurs under ambient conditions with no additional chemicals. The laser used for this process is low-power, continuous, and affordable, making it a rapid and cost-effective alternative suitable for large-scale applications. This makes LIG an accessible and scalable technique for fabricating graphene-based materials for applications in energy storage, sensors, and flexible electronics.

While many reports present approaches for integrating active materials into graphitic matrices, they often rely on the use of presynthesized carbon and nanomaterials. For example, previous studies have shown that mixing premade Si nanoparticles with LIG precursors can create silicon-graphene anodes for lithium-ion batteries (LIBs).^23,24^ However, the use of nanoparticles is often undesirable due to high costs and processing difficulties. Therefore, a key challenge is to develop processes that can impart nanoscale electrochemical advantages by using raw inexpensive micropowders through simple and scalable fabrication routes.

A particularly relevant example in Li-ion batteries is the use of Si and silicon monoxide (SiO) microparticles instead of their nanoparticle counterparts, which are currently more prevalent. Micro-Si powders offer several advantages, including cost-effectiveness and sourcing from low-grade material, metallurgical-grade materials, or waste streams.^29^ However, their application in Li-ion batteries has been hindered by several challenges such as increased pulverization, mechanical instability, poor lithium-ion diffusion, and low conductivity, all of which significantly limit their electrochemical performance.

Significant efforts have been made to adapt micro-Si for battery applications. Some strategies focus on utilizing methods that partially mitigate these issues such as various types of chemical etching,^30,31^ use of specific alloys,^32^ core–shell structuring,^33^ and electrolyte engineering.^34^ However, these approaches address only a portion of the existing challenges, namely the performance issues, and are either highly complex, hazardous, costly, and time-consuming, or require some form of other prefabricated nanomaterial, e.g., a reagent or catalyst. Consequently, there is a critical need for innovative methods that enable the direct and effective fabrication of diverse active nanocomposites from bulk precursors for advanced energy storage systems.

Here, we present a different approach, termed ″laser milling”, which leverages the ultrafast photothermal graphitization of the laser-induced graphene (LIG) process. This single-step, low-power, laser-triggered method simultaneously reduces bulk microparticles of various active materials, including Si and SiO, to the nanoscale while concurrently transforming low-cost, readily available carbon precursors into a highly conductive, porous, graphene-like carbon matrix that embeds the active nanomaterials. This yields a composite that offers the processing ease of microparticles and the overall performance of nanoparticles anchored within a structural graphitic support.

Thus, “laser-milling” and LIG formation proceed as simultaneous, interdependent processes, resulting in a monolithic nanoparticle/LIG (NP/LIG) composite. In our investigation, the principal constraint on material selection was found to arise from the irradiation temperature: laser graphitization can generate extremely high localized temperatures (>2000 K) within milliseconds, and depending on the thermal properties of the target material, ultrafast photothermal kinetics may trigger explosive boiling and violent ejection of molten material, resulting dispersion of nanodroplets that subsequently resolidify as spherical nanoparticles.

It is worth noting that several laser-based methods have been developed for top-down nanoparticle synthesis with high-power pulsed laser ablation of ultrapure metal targets being particularly prevalent. However, despite its ability to produce high-purity, contaminant-free nanomaterials without chemical reagents, these approaches suffer from major drawbacks, including substantial energy consumption, reliance on costly laser systems, and limited scalability, in addition to issues such as excessive local heating, uncontrolled plasma dynamics, and material degradation.^35–38^

In contrast, our “laser-milling” method employs a low-cost, continuous, low-power (2.8 W) blue diode laser. The process is dominantly driven by the interaction between the laser and the graphene precursor, where ultrafast photothermal graphitization simultaneously forms the conductive matrix and reduces the size of microparticles to the nanoscale, thereby enabling nanoparticle formation and in situ integration into a graphitic framework with far greater scalability and cost-effectiveness.

Here, we demonstrate our “laser-milling” approach for the synthesis of three types of NP/LIG composites, using phenolic resin (PR) as the carbon precursor and micropowders of silicon, silicon monoxide, and magnesium as active materials. This strategy highlights the versatility of the method, producing a broad range of nanocomposites for diverse applications, requiring deeply integrated structures. Thus, the presented study establishes “laser-milling” as a novel top-down synthesis method for graphene-embedded nanoparticle synthesis from simple microparticle/resin precursor blends as a scalable, single-step, low-power process with broad applications in energy storage and other technologies.

## Materials and Methods

### Sample Synthesis

Phenolic resin (PR, Polyols & Polymers Pvt. Ltd) was dissolved in anhydrous ethanol to obtain a 50 wt % solution. 325 mesh silicon (Si), 325 mesh (<44 μm) silicon monoxide (SiO), 125 mesh (<125 μm) porous alumina (Al_2_O_3_), and magnesium powder (Mg) (Sigma-Aldrich) were added in 5 wt % and dispersed in the PR solution using a vortex mixer. No other dispersion methods were used to avoid size reduction due to the use of a dispersion apparatus (e.g., sonicator probe).

The as-prepared slurry was coated onto a 316 mm stainless-steel disk (0.5 mm) without binders or additives. Deposition was performed using either doctor-blade coating (50 μm height) or a two-layer spin-coating process (300 rpm for 10 s followed by 1000 rpm for 20 s). For spin-coated films, each layer was dried at 40 °C for 2 min. Following the final application, all films were dried at 60 °C for 1 h under vacuum.

The resulting coated steel disk was then irradiated using continuous visible laser irradiation (2.8 W, 450 nm wavelength, Zmorph Fab) at a hatch spacing of 0.1 mm and a defocusing height of 5.5 mm (spot size approximately 50 μm). The rastering speed was set at 4 mm/s, which correlates to a dynamic fluence of 1.40 J/mm^2^, as calculated by the equation described by Stamatin et al.^39^ using the equation

$$F=\frac{P}{\mu \times D}$$

where *F* is the dynamic fluence (J/cm^2^), *P* is the laser power (W), μ is the rastering speed (cm/s), and *D* is the spot diameter (cm). Each sample was irradiated twice. To adjust the fluence, the scanning speed was varied accordingly.

Control anodes were prepared using a PVDF binder (Nanografi) and carbon black (Super P, Nanografi), mixed with SiO microparticles in an 80:10:10 weight ratio, respectively, for an approximate loading of 1 mg/cm^2^. The resulting slurry was cast onto a current collector using a doctor blade with a 70 μm wet film thickness.

To prepare additional control anodes for comparative study, SiO powder was processed via ball milling. The powder was loaded into a 50 mL stainless-steel jar with a ball-to-powder weight ratio of 30:1. After sealing in an argon-filled glovebox, the jar was milled for 20 h at 600 rpm. This mechanical approach was used solely to establish a performance baseline; it is not required for the proposed laser-based methodology, which achieves micro- to nanoparticle reduction in situ during a single step.

### Material Characterization

High-resolution scanning electron microscopy (HRSEM) imaging and energy-dispersive X-ray spectroscopy (EDS) were conducted using a Zeiss GeminiSEM 300 system. Transmission electron microscopy (TEM), scanning transmission electron microscopy (STEM), and further EDS analyses were conducted using a Themis Z G3 system (ThermoFisher). X-ray photoelectron spectroscopy (XPS) measurements were conducted using an ESCALAB QXi X-ray Photoelectron Spectrometer Microprobe using an Al Kα source. Raman spectroscopy measurements were conducted using a LabRAM HR Evolution Confocal Raman Microscope (HORIBA) with a 532 nm wavelength laser. Powder X-ray diffraction (PXRD) analysis was conducted by using a Bruker D8 Discover diffractometer with a Cu Kα source. Peak assignments were identified using the Inorganic Crystal Structure Database (ICSD) and the relevant literature. Particle size histograms were acquired by image analysis using ImageJ software.^40^

### Electrochemical Analysis

The electrochemical evaluation of the SiO/LIG sample was performed using a Neware BTS battery test system (Neware LTD). A SiO/LIG half-cell was assembled in an inert glovebox (<0.1 ppm of O_2_, H_2_O) using the as-prepared sample as the working electrode, versus a lithium metal (S4R) counter electrode. The electrodes had an areal mass loading of approximately 0.5 mg/cm^2^. The electrolyte was 45 μL of 85% 1 M LiPF_6_ in 1:1 ethylene carbonate/diethyl carbonate (EC:DEC; Sigma-Aldrich) and 15% fluoroethylene carbonate (FEC; Solvay-Fluor). The separator used was a Celgard 2325. The assembled cells were kept at 25 °C during the cycling and C-rate tests. To ensure a rigorous experimental control comparison, ball-milled counterparts were prepared using an identical loading weight of the ball-milled nanoparticles and fabricated according to the same protocol.

The full cell was prepared by using a lithium iron phosphate (LFP) cathode (Nanografi). The cathode slurry consisted of LFP, carbon black (Super P, Nanografi), and poly­(vinylidene fluoride) (PVDF) binder dispersed in *N*-methyl-2-pyrrolidone (NMP) solvent at a weight ratio of 90:5:5, corresponding to an areal loading of approximately 0.5 mg/cm^2^. The cell was cycled between 2.2 and 3.8 V.

## Results and Discussion

### Synthesis Process and Mechanism Overview

The “laser-milling” process developed in this study represents a new approach to top-down nanoparticle synthesis within a laser-induced graphene (LIG) matrix. As illustrated schematically in Figure 1a, the process begins with the preparation of a precursor ink consisting of phenolic resin (PR) dissolved in ethanol. PR was selected as the precursor because it is cost-effective, dissolves readily in ethanol, and has been shown to form high-quality LIG in various applications.^23,24,27,28^ Ceramic and metallic microparticles, such as magnesium (Mg), silicon monoxide (SiO), silicon (Si), or alumina (Al_2_O_3_), were homogeneously dispersed into the PR solution, and the resulting ink is then coated directly onto a substrate.

**Figure 1:**
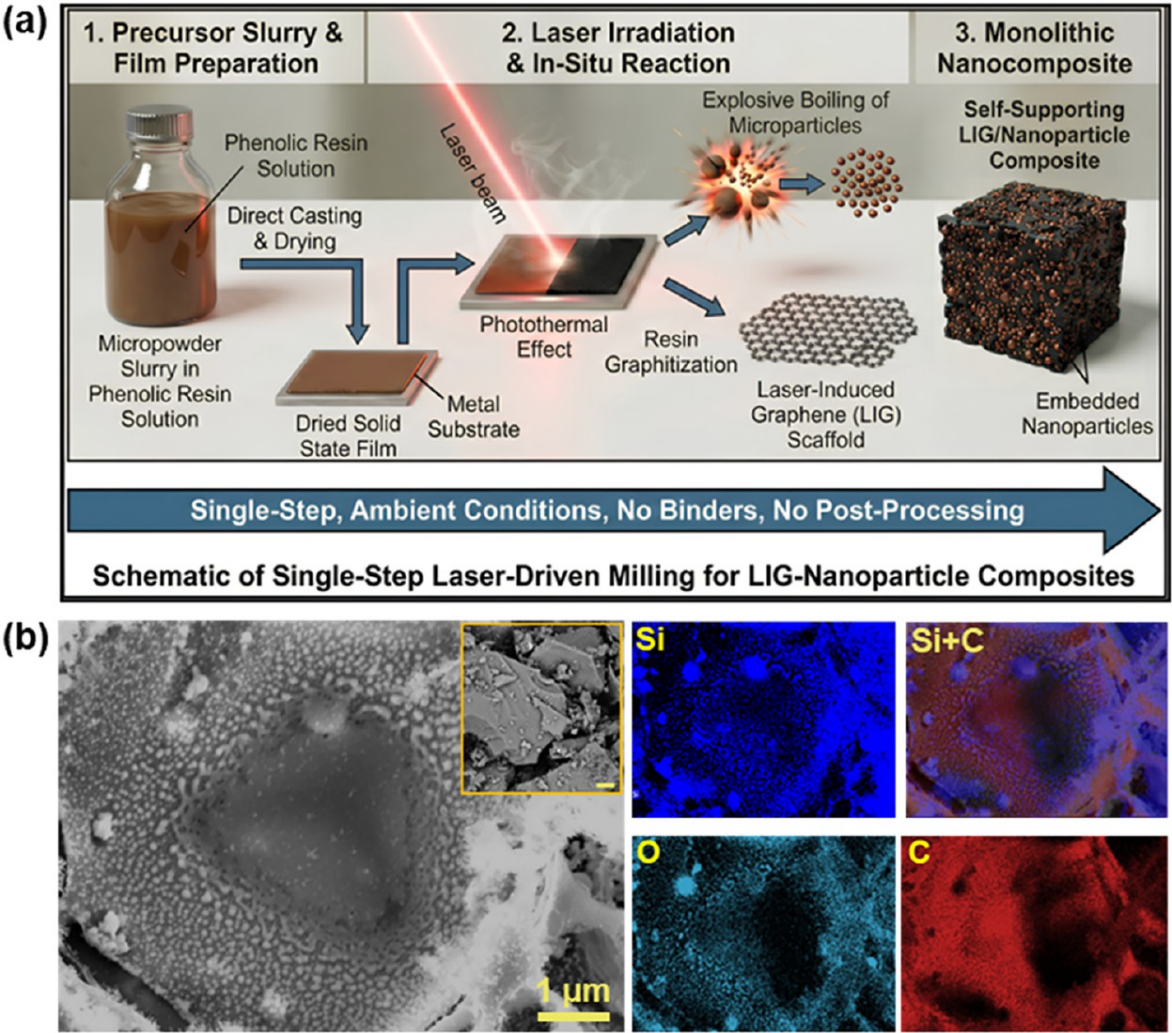
(a) Schematic representation of the single-step synthesis of NP/LIG nanocomposites by laser irradiation of a micropowder/phenolic resin precursor blend. (b) SEM-EDS imaging of a single Si microparticle “laser-milled” and its supporting LIG matrix, leaving a crater surrounded by nanoparticles. The inset of the SEM image shows the original microparticles at a scale bar of 1 μm.

The choice of substrate provides versatility in applications, accommodating smooth, rough, or porous surfaces such as stainless-steel current collectors, glass slides, and metal foils. In this study, the ink was applied through techniques such as spin-coating or doctor-blade coating, followed by drying under controlled conditions. Subsequently, the dried film underwent rastering with low-power (2.8 W) continuous visible laser irradiation at 450 nm, creating a composite material where the precursor matrix transforms into a LIG framework.

The basis for the proposed mechanism for the formation of the various NP/LIG composites is primarily based on the well-established process of LIG formation. However, a comprehensive and separate analysis of the thermodynamics and kinetics governing the proposed explosive boiling micro- to nanoparticle reduction mechanism is provided in the Mechanistic Discussion section.

In short, the absorption of photon energy by the matrix triggers photothermal or photochemical reactions, resulting in the rapid pyrolysis of the carbon precursor. While the absorption of laser energy by the microparticles alone is insufficient to induce the complete breakdown of large particles, as will be demonstrated, the pyrolysis process itself generates highly energetic conditions, characterized by intense localized heating (found to exceed 2000 K), bond cleavage, outgassing, and elevated pressures. These extreme conditions facilitate the formation of LIG, as they thermodynamically favor the nucleation of graphene nanodomains.^41,42^ Subsequently, these conditions also facilitate the reduction of microparticle precursors to nanoparticles.

Fundamentally, we propose that “laser-milling” operates through three concurrent phenomena, two of which arise directly from the LIG synthesis process: (1) the generation of extreme localized temperatures (>2000 K) via ultrafast photothermal and/or photochemical reactions, (2) the evolution of gases, primarily CO and H_2_, from the precursor matrix, and (3) the explosive boiling of microparticles under these nonequilibrium conditions. The laser-induced graphitization of the polymer precursor is central to this environment: it produces ultrafast heating on a millisecond scale, creating thermal shocks that can superheat the embedded microparticles. Under such rapid heating, there is insufficient time for conventional melting or boiling to occur. Instead, the particles most likely undergo explosive boiling, a catastrophic phase transition in which superheated material vaporizes violently once nucleation barriers are exceeded.^43,44^ The precise mechanism discussion is expanded further in the manuscript.

Notably, the combination of ultrahigh temperatures, millisecond kinetics, and stabilization within a graphitic matrix distinguishes this process from conventional fragmentation routes and accounts for the efficiency and uniformity of the nanoparticle generation. At the same time, nanoparticles are directly embedded into the LIG framework, producing a monolithic self-supporting composite with exceptional structural and functional properties.

SEM imaging and EDS elemental mapping (Figure 1b) provide initial direct evidence of the mechanism, revealing microscale craters surrounded by spherical nanoparticles on the LIG substrate. To the best of our knowledge, this is the first demonstration that such a mechanism governs size reduction during ultrafast photothermal graphitization, enabling the single-step transformation of microparticles into uniform nanoparticles.

### Structural and Morphological Characterization

Initial morphological characterization of the various NP/LIG composites was conducted by using SEM and TEM, providing valuable insights into the synthesis processes occurring during the lasing process.

The materials selected in this study were chosen due to their distinct thermal and chemical properties. Mg is a highly reactive metal that readily oxidizes in ambient conditions, which results in a thin MgO coating even after minimal exposure. SiO is a metastable amorphous phase, thermodynamically more stable than Mg under ambient conditions, and in fact consists of a disproportionated mixture of Si and SiO_2_. Pristine Si and Al_2_O_3_, on the other hand, are both chemically stable materials with high melting points (1414 and 2072 °C, respectively). This variation enables the investigation of the different composite materials that can be obtained from distinct pristine precursors.

As described earlier, all powders were dispersed in the same LIG precursor material, which has also been employed in several recently published studies.^24,27,28^ To provide a clear baseline for comparison, pristine microparticles were characterized alongside the resulting composite materials; images of these pristine particles are displayed in Supporting Figure S1. Overall, all composite samples share a similar LIG porous backbone, into which the particles are embedded, as seen in SEM imaging in Figure 2.

**Figure 2:**
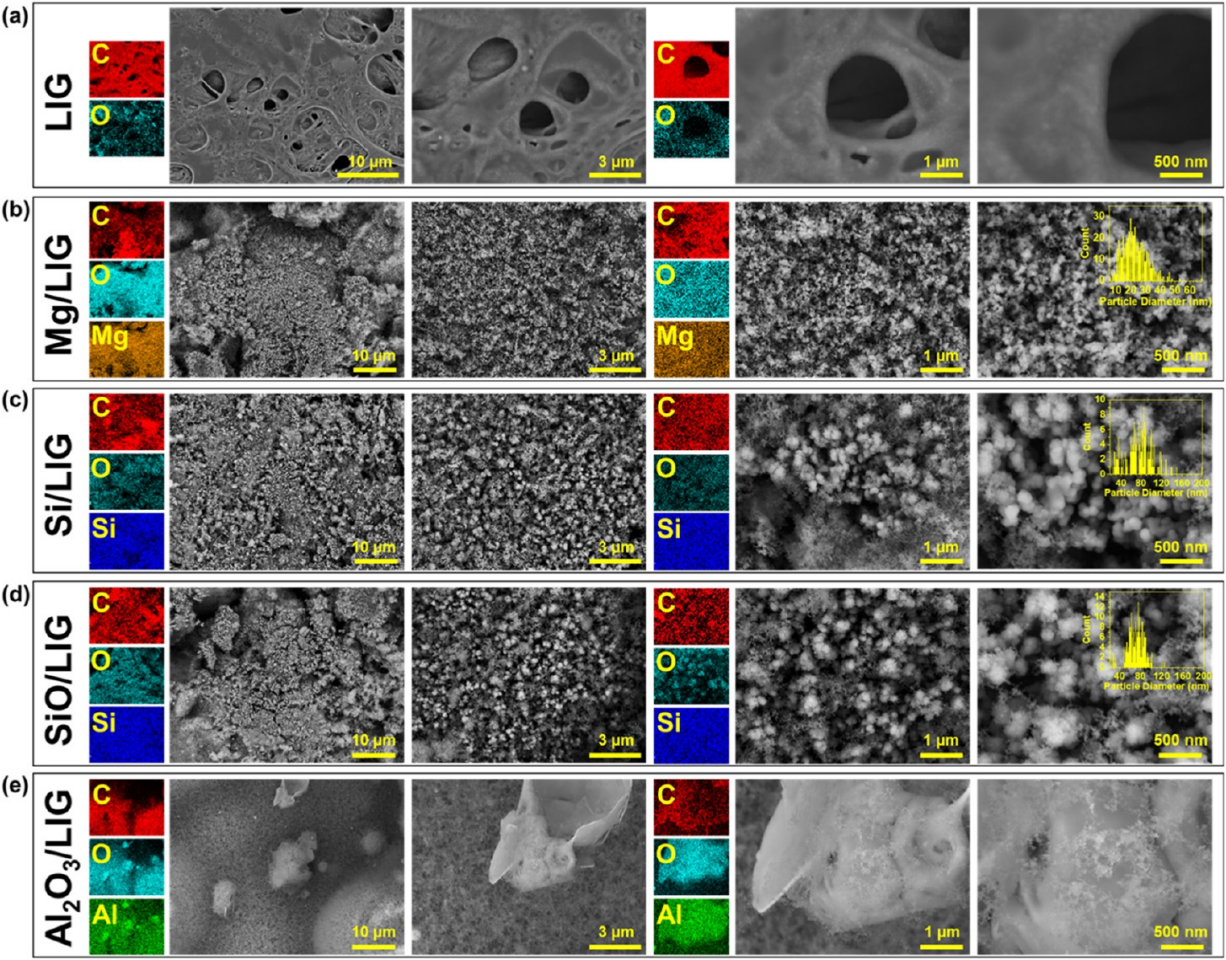
SEM imaging of (a) pristine LIG and microparticle-derived (b) Mg/LIG, (c) Si/LIG, (d) SiO/LIG, and (e) Al_2_O_3_/LIG composites with the corresponding EDS analysis. Insets are histograms describing the particle size distribution of the nanoparticles in the image where nanoparticle formation occurred and is relevant.

Figure 2a depicts a control sample of the phenolic resin precursor without microparticles, laser irradiated under conditions identical to those for the microparticle/resin blends. SEM and EDS imaging of the resulting LIG confirm that no nanoparticles are formed during the photothermal conversion of the neat resin. This result is essential to demonstrate that nanoparticle formation is specific to the presence of microparticles and is not an inherent byproduct of the LIG formation process or the laser irradiation parameters.

Figure 2b–e shows SEM/EDS images with increased magnification of the NP/LIG nanocomposites. A backscattered electron detector was used for SEM imaging which is more sensitive to the atomic mass of the material and gives ample contrast between the carbon matrix and synthesized nanoparticles.^45^

Across all samples, the microparticle precursors are reduced to nanoscale sizes, typically ranging from 10 to 300 nm, with the vast majority in the sub-100 nm range, as can be seen via the histogram insets in Figure 2, which were produced by image analysis. The graphitization process also induces significant porosity in the carbon matrix, attributed to the outgassing of volatile species during laser irradiation.^45^ This porosity, along with the formation of a distinct carbon backbone, facilitates uniform dispersion of nanoparticles throughout the matrix.

Supporting Figure S2 provides cross-sectional SEM imaging of the composites, confirming that this porosity and the laser-driven transformation extend throughout the entire volume depth. This is a critical observation, as it demonstrates that the reaction is complete and that no residual, unirradiated insulating layers remain at the bottom of the samples. This consistent porosity, along with the formation of a distinct carbon backbone, facilitates the uniform dispersion of nanoparticles throughout the matrix.

The effect of laser fluence on NP size and density was investigated by using SiO as a representative material. Based on established optimization studies for this precursor and laser system,^23,27^ a fluence range of 0.35 to 5.6 J/mm^2^ was selected to ensure high-quality formation. Within this optimized window, particle size and distribution remained largely consistent, although minor variations in porosity were observed. Notably, at the lower threshold of 0.35 J/mm^2^, a significant reduction in particle density occurred. These results, detailed in Supporting Figure S3, indicate a wide growth regime for the synthesized NPs.

SEM analysis presented in Figure 2b reveals that the Mg/LIG composite is composed of nanoparticles on the porous carbon backbone. This porosity arises from the outgassing of volatile compounds during the laser-driven graphitization process. Nanoparticles are uniformly distributed throughout the porous carbon framework, indicating a strong integration within the matrix. The corresponding histogram inset shows a mean particle size of approximately 30 nm. EDS mapping confirms the presence of Mg in the particles themselves, while oxygen is detected at high concentrations around the particles. This suggests significant surface oxidation of the Mg nanoparticles, along with partial oxidation of the LIG backbone.

As a positive control, the Mg micropowder was irradiated under identical conditions without phenolic resin. As shown in Figure 3a, no particle size reduction occurred. Although minor surface effects likely due to slight photothermal heating or oxidation were observed, the overall morphology remained unchanged. The lack of particle reduction in the absence of the resin indicates that the mechanism for size reduction is specifically driven by the interaction during the carbon graphitization into LIG.

**Figure 3:**
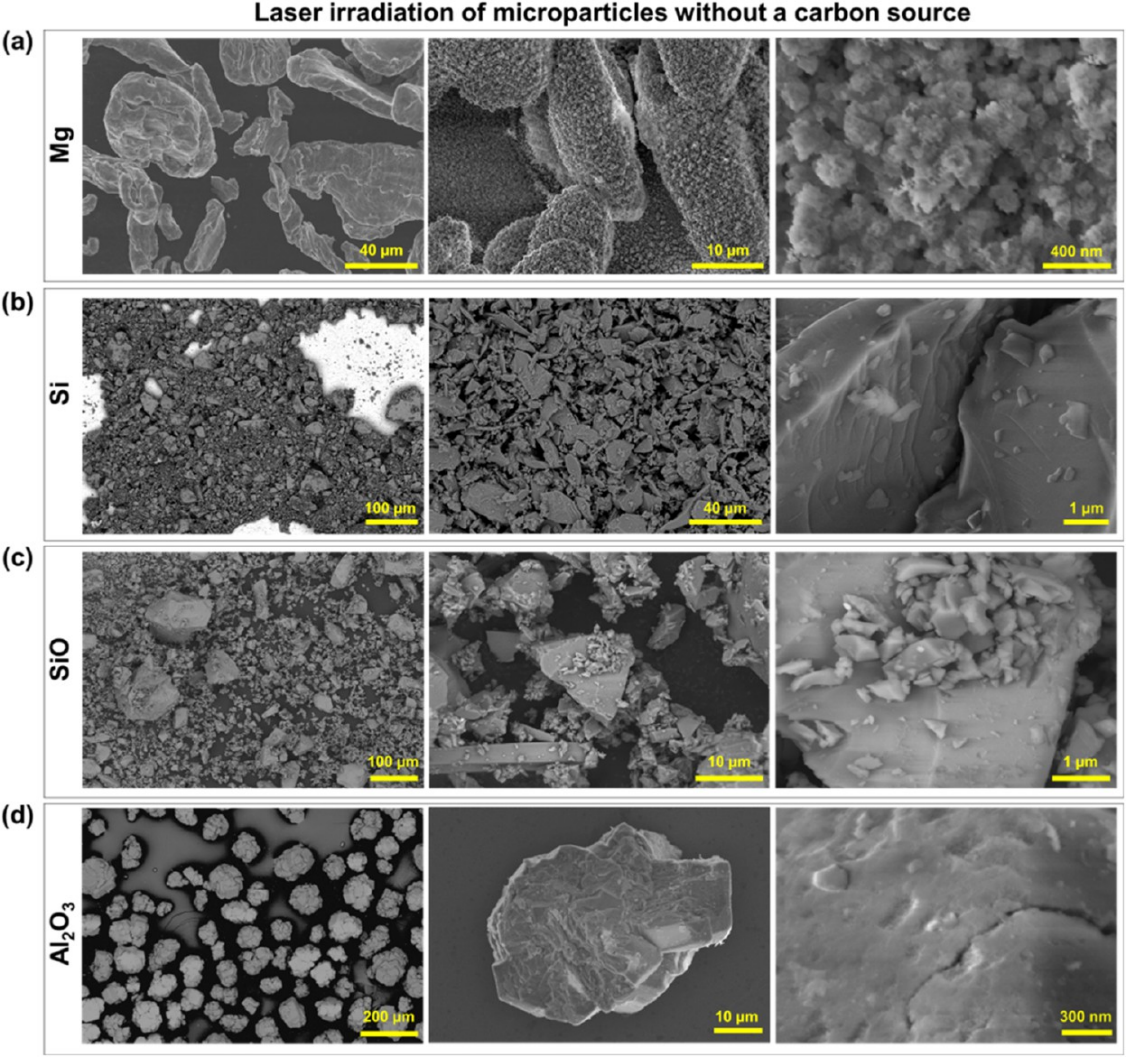
SEM imaging of (a) Mg, (b) Si, (c) SiO, and (d) Al_2_O_3_ microparticles following visible laser irradiation without a carbon source.

The TEM image displayed in Figure 4a of the Mg/LIG sample shows base particles ranging from 30 to 50 nm, with high crystallinity observed. Surrounding the MgO particles, the supporting carbon matrix is clearly visible and is measured in the high-resolution inset, measuring the LIG lattice spacing, providing evidence of the concomitant synthesis of nanoparticles and the LIG matrix during laser milling. This intimate interaction between MgO and the graphitic carbon framework underscores the effectiveness of the laser milling process in achieving a homogeneous, integrated structure. The graphene mechanically anchors the Mg/MgO particles and enhances the composite’s conductive properties.

**Figure 4:**
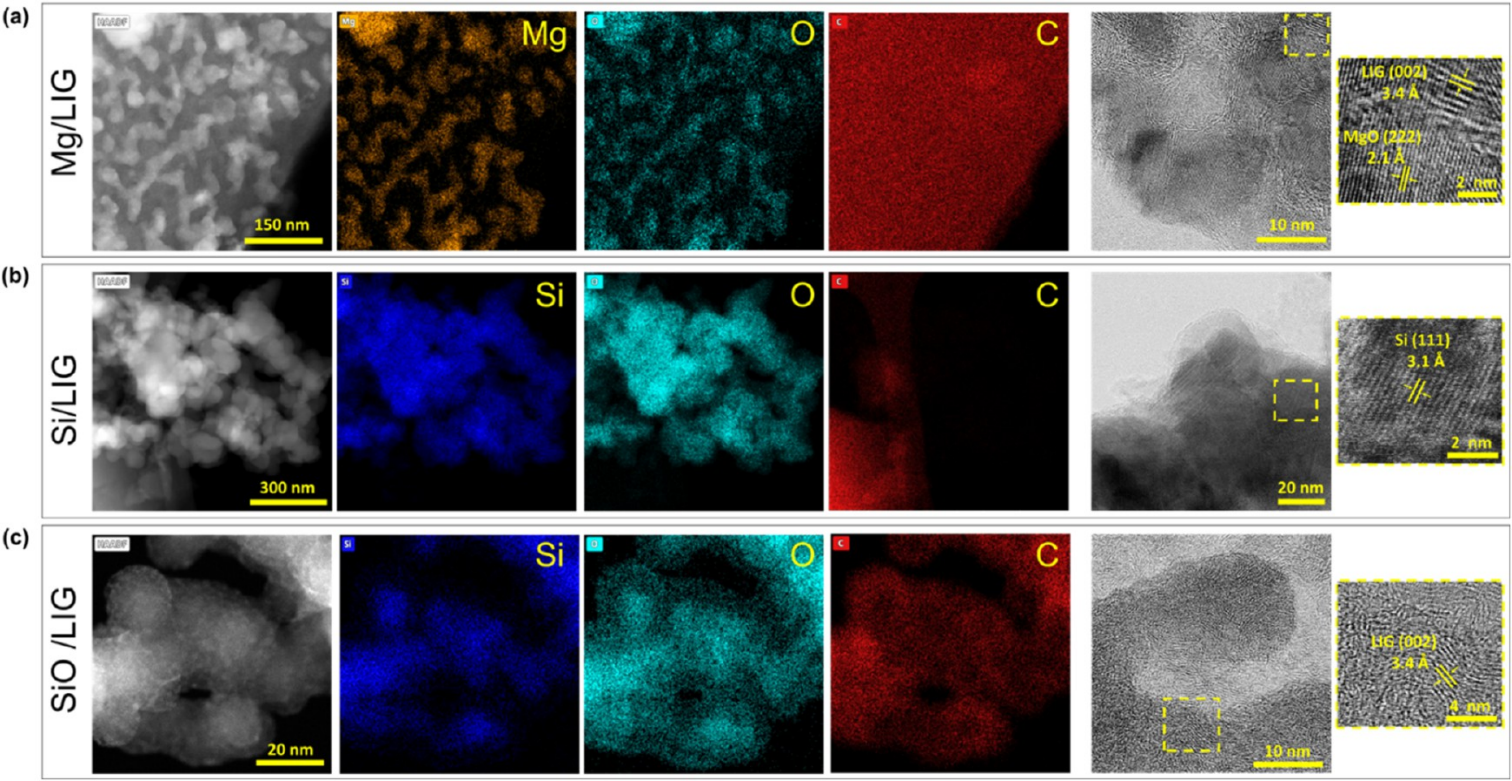
TEM and EDS images of microparticle-derived “laser-milled” nanoparticles in samples of (a) Mg/LIG, (b) Si/LIG, and (c) SiO/LIG, showing the nanoparticles embedded within graphitic carbon. The insets depict high-resolution imaging of the lattice spacing for each sample.

The electron microscopy results are further corroborated by the structural spectra of the Mg/LIG as shown in Figure 5a. Comparative analysis of the XRD spectra for pristine Mg and the Mg/LIG composite reveals that while both spectra exhibit strong peaks for metallic Mg, the Mg/LIG sample also shows additional peaks that correspond to MgO. This indicates that some oxidation of the original Mg has occurred; however, it is limited and not exhaustive.

**Figure 5:**
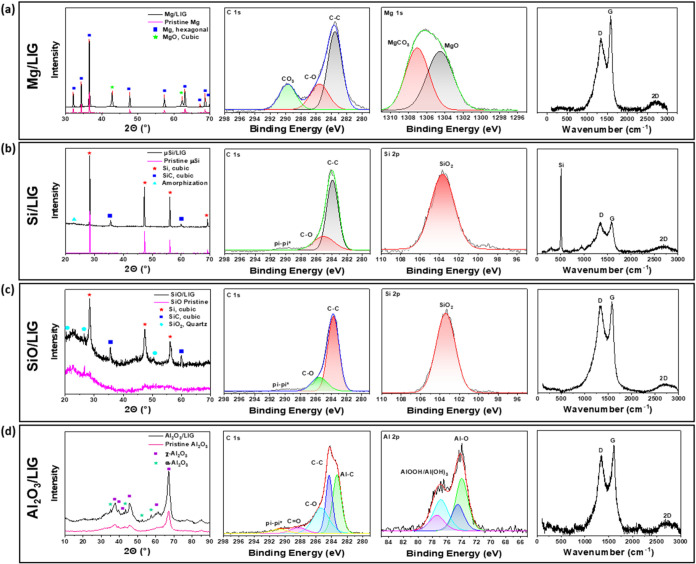
XRD, XPS, and Raman spectra of (a) Mg/LIG, (b) Si/LIG, (c) SiO/LIG, and (d) Al_2_O_3_/LIG.

XPS data of Mg/LIG displayed in Figure 5a further illuminate surface details that are challenging to capture in XRD measurements, especially through deconvolution of the C 1s and Mg 1s regions. In the C 1s spectrum, a notable carbonate peak appears at 290.0 eV, while in the Mg 1s spectrum, peaks corresponding to MgO and MgCO_3_ are present at 1304.5 and 1307 eV, respectively.

Additionally, the XPS C 1s spectra of Mg/LIG in Figure 5a display a high proportion of C–C bonds, which is consistent with high-quality, largely nonoxidized LIG. Additionally, XPS analysis of the initial phenolic resin precursor, shown in Supporting Figure S4, reveals a high sp^3^ hybridized carbon content, consistent with the highly cross-linked structure of the polymer before its conversion to graphene. This C–C bonding structure, observable in the Raman spectrum as well, reflects the transformation of the carbon matrix, reinforcing the integrity and conductive potential of the LIG scaffold.

Raman spectroscopy analysis of Mg/LIG in Figure 5a provides additional insights into the nature of the carbon within the composites. The presence of a very strong G peak indicates successful graphitization, confirming the conversion of the carbon precursor to a graphitic structure. The D peak, while also pronounced, is somewhat less intense than the G peak, suggesting a moderate level of defects or a high density of edges. This observation implies numerous small graphitic regions, where the edges contribute to the structural characteristics of the graphene. Additionally, the broad 2D peak in the spectrum reveals that the graphene is a multilayered, relatively disordered structure with extensive nanoscale regions. The graphitic carbon backbone is essential for maintaining structural integrity and ensuring even nanoparticle dispersion throughout the matrix, resulting in a monolithic, conductive, and self-supporting structure with high stability and moderate oxidation.

The SEM images at various magnifications of the Si/LIG sample in Figure 2c reveal a carbon backbone composed of a LIG within which Si nanoparticles are embedded. The corresponding histogram inset shows a mean particle size of approximately 80 nm, with some particles measuring significantly smaller. The EDS image in Figure 2c supports these observations, showing a strong Si signal covering the area. These results indicate that the mechanism is effective for Si as well as Mg, demonstrating the versatility of the process.

The observed porosity, as in the other samples, results from rapid outgassing during the pyrolysis of the carbon precursor. The dynamic interplay among melting, fragmentation, and rapid cooling is evident in the particle morphology, showing a gradual transition into spherical nanoparticles. This variability underscores the concomitant nature of the laser-milling process, encompassing initial laser-induced heating, pyrolysis-driven outgassing, and subsequent particle fragmentation.

This specific distribution of silicon provides critical insights into the “laser-milling” mechanism, resulting from a possible explosive boiling effect.^43^ We propose that the heat generated by the laser within the carbon matrix is sufficient to melt the microparticles and superheat them. This possibly leads to explosive boiling, where the molten material is ejected as nanodroplets.^44,47^ These ejected materials rapidly deposit onto surrounding surfaces, as the cooler environment facilitates their condensation, thereby transforming the large microparticles into spherical nanoparticles and nanostructured materials.

The LIG backbone, formed from the graphitization of phenolic resin, stabilizes the dispersed silicon nanoparticles and maintains the structural integrity of the composite. Beyond its structural role, this conductive carbon matrix ensures effective electron transport, making the material especially suitable for electrochemical applications, such as battery anodes. The homogeneous integration of silicon nanostructures into the LIG framework underscores the monolithic nature of the composite, enhancing both its functional and structural properties.

As a positive control, SEM imaging of pristine Si micropowder in Figure 3b irradiated without a carbon source further demonstrates the necessity of carbon graphitization for the laser milling process. Si particles exposed to lasing under identical conditions remained unchanged, confirming that the reduction mechanism is dependent on the interaction with the resin during its conversion to LIG.

Detailed TEM analysis, presented in Figure 4b, shows Si nanoparticles dispersed on the carbon support. Moreover, high-resolution STEM lattice measurements further support that the silicon remained predominantly in the Si^0^ state. We attribute this to the local microenvironment, which is not simply inert but instead mildly reducing, thereby suppressing oxidation.

XRD (Figure 5b) analysis of the Si/LIG samples further confirms the retention of a substantial crystalline structure in the silicon. Comparison of the Si/LIG composite with pristine Si micropowder shows some important distinctions. Small peaks corresponding to silicon carbide (SiC) are present, along with a slight increase in intensity at lower angles, manifesting as a broad peak, which suggests the presence of amorphous silicon species. As XRD primarily reflects the bulk crystalline properties of a material, this technique provides reliable information regarding the crystalline and amorphous species of silicon within the composite, complementing the detailed surface information gained from XPS.

The XPS (Figure 5b) analysis of the Si/LIG sample reveals a contrast between the surface and bulk properties of silicon. Surface-level silicon appears almost entirely oxidized, as indicated by a single Si 2p peak corresponding to SiO_2_. This oxidation is confined to silicon directly at the surface, while the bulk retains its distinct properties. Additionally, the C 1s spectrum primarily shows sp^2^ hybridized C–C bonds and distinctive pi-pi* loss interactions, further supporting the transformation of the carbon precursor into a graphene-like structure within the composite.

Moreover, Raman spectroscopy analysis (Figure 5b) of the Si/LIG sample provides another confirmation of the crystalline silicon (c-Si) structure, showing a sharp peak at 507 cm^–1^, which is associated with c-Si. Notably, this peak is shifted by approximately 13 cm^–1^ from its typical position at 520 cm^–1^. This shift can be attributed to phonon confinement effects commonly observed in nanocrystalline silicon, as well as the presence of amorphous silica, as indicated by XPS. Both factors contribute to the observed deviation, reinforcing the presence of nanoscale and partially amorphous silicon within the Si/LIG composite. Additionally, the formation of characteristic high-defect-rate multilayered LIG is confirmed by the presence of D and G peaks with an intensity ratio of ∼1, and a wide 2D peak.

For the SiO/LIG sample, SEM-EDS imaging (Figure 2d) reveals the same highly porous LIG network, characterized by prominent voids. EDS mapping confirms silicon and oxygen as the dominant elements. The O:Si atomic ratio, approximately 1.1, suggests minimal oxidation, with most silicon retaining or only slightly altering its original oxidation state. The carbon scaffold plays a critical role in providing a stable framework for the nanoparticles, ensuring structural coherence despite the extensive porosity introduced by the lasing process. Evidence of surface oxidation, as opposed to bulk oxidation, is further supported by XPS, XRD, and TEM analyses, which will be discussed in the following paragraphs. An additional positive control in Figure 3c depicts the SiO microparticles irradiated without a carbon source; similar to the other microparticles, these show no discernible or noticeable reduction in size.

TEM imaging (Figure 4c) reveals SiO base particles approximately 15 to 20 nm in diameter, clustering along the LIG backbone. These particles remain predominantly amorphous, consistent with the starting SiO material. EDS mapping confirms that silicon oxides likely dominate the particle surfaces, with oxygen forming a shell around the silicon cores. The carbon distribution closely follows this pattern, suggesting the presence of a thin carbon layer coating the particles. This carbon layer likely plays a critical role in stabilizing the SiO nanoparticles and ensuring their interaction with the LIG matrix. Despite their amorphous nature, the SiO particles are fully integrated within the LIG backbone, which is key to the overall uniformity and functionality of the SiO/LIG composite.

Notably, the differences in crystallinity between the Si/LIG and SiO/LIG samples underscore the influence of the starting material on the final composite structure. The rapid cooling inherent to laser milling further accentuates these differences, promoting nanocrystallite formation in crystalline Si, while preserving the amorphous state in the SiO precursors. These findings highlight the versatility of the laser milling process in tailoring nanoparticle properties based on the precursor composition and process conditions.

The XRD (Figure 5c) spectra of the pristine SiO sample display a very weak peak associated with crystalline Si, though the spectrum is largely dominated by a broad signal in the low 2θ region, characteristic of amorphous silicon materials in various oxidation states. Following the laser graphitization process, distinct peaks corresponding to Si and silicon carbide (SiC) emerge. While these crystalline materials were undetected in localized methods such as TEM and SEM, the bulk statistical sensitivity of XRD to crystalline structures confirms their formation as a result of the lasing process. This formation is likely attributed to carbothermal reactions or thermal disproportionation of SiO during laser treatment. XPS analysis in Figure 5c further clarifies the oxidation states, showing a single peak for SiO_2_ in the Si 2p region. Additionally, in the C 1s region, there is an increase in the proportion of C–C bonds relative to C–O bonds when compared to the original phenolic resin precursor (Figure S1). This shift suggests a graphitic transformation in the carbon structure, aligning with the expected changes brought about by the lasing process.

Finally, the Raman spectrum of SiO/LIG in Figure 5c again shows a highly defective, multilayered LIG, as pronounced by the intensity ratio of the D and G peaks and a wide 2D peak. The absence of a peak near 520 cm^–1^ that can be attributed to crystalline Si is notable and in contrast to the results by the XRD. This is likely due to the small presence of crystalline Si, too low to be detected by Raman spectroscopy.

As shown in Figure 2e, the alumina in the Al_2_O_3_/LIG composite underwent only minor changes during the lasing process. While the LIG backbone derived from the phenolic resin precursor is clearly visible, Al_2_O_3_ remained as large particles ranging from ∼5 to 100 μm in diameter. The pristine Al_2_O_3_ as purchased is shown in Supporting Figure S1, while Figure 3d displays the same material after laser irradiation in the absence of carbon. In both cases, the particles remained unchanged, indicating that the lasing process has no effect on Al_2_O_3_ regardless of whether carbon is present. This stark difference between Al_2_O_3_ and Si is attributed to the significantly higher melting point of alumina and the higher enthalpy of fusion. The minimal temperatures in the LIG synthesis are generally considered around 2000 K, as calculated by simulations,^46,48^ meaning it is likely that these temperatures are not enough to fully heat large alumina particles and induce explosive boiling.

However, XRD analysis of Al_2_O_3_/LIG shows that some degree of phase change does happen. As shown in Figure 5d, the original precursor material measured by XRD is shown to be slightly amorphous χ-Al_2_O_3_, which is a metastable phase. Following the lasing process, peaks that can be attributed to α-Al_2_O_3_ were measured alongside the original χ-Al_2_O_3_, which strongly suggests that a small but significant amount of the alumina underwent surface phase changes, made possible by the high temperatures produced by the graphitization.

The XPS analysis of the Al_2_O_3_/LIG sample (Figure 5d) highlights the highly reactive and reducing atmosphere generated during the graphitization of the phenolic resin precursor. In the C 1s region, the spectrum reveals multiple deconvoluted peaks: a prominent C–C peak, a significant C–O peak, a smaller C=O peak, and a distinct tail attributed to pi-pi* loss interactions. These features correspond to the LIG backbone structure, characterized as a partially oxidized, graphene-like material. Notably, a distinct peak at ∼283 eV, attributed to Al–C bonds, was observed, indicating substantial interaction between aluminum on the alumina particle surfaces and carbon during LIG synthesis.

In the Al 2p orbital, as shown in Figure 5d, a high degree of surface hydroxylation of the alumina particles is evident. Peaks near ∼77 eV, associated with AlOOH and Al­(OH)_3_, were detected alongside Al–O peaks at ∼74 eV, reinforcing the evidence for a reducing atmosphere produced during laser irradiation of the phenolic resin. This is further supported by the decreased amount of AlOOH/Al­(OH)_3_ in XPS measurements following ion sputtering of the sample (Supporting Figure S5), showing that the alumina is less reduced in deeper regions.

The Raman spectra (Figure 5d) further confirm the quality of the LIG. The G band is slightly more intense than the D band, indicating a moderate level of structural defects, while the broad 2D band confirms that the LIG is multilayered. These results demonstrate that the carbon precursor was fully transformed into LIG, even in the absence of the “laser-milling” size reduction process. Thus, while the temperatures required for LIG formation were reached, they were not sufficient to drive the dimensionality reduction process for Al_2_O_3_, highlighting the limitations of the process, although versatile.

### Applications as a Battery Anode Material

To demonstrate the applicability of the proposed nanoparticle production method, a Li/Li^+^–SiO/LIG half-cell was fabricated. This was achieved by mixing pristine SiO micropowder with an LIG precursor (phenolic resin), followed by laser processing at an energy density of 1.4 J/mm^2^. This proof-of-concept cell highlights the potential of the developed process for scalable and cost-effective electrode fabrication. Given that Li-ion batteries are the dominant technology for electrical energy storage, powering everything from consumer electronics and electric vehicles to grid-scale systems, even modest reductions in manufacturing cost or improvements in efficiency can have a substantial technological and economic impact.

When considering anode materials for lithium-ion batteries (LIBs), any increase in the gravimetric capacity is paramount, as it allows for smaller, lighter batteries. The most common anode material, by far, is graphite, which has ∼372 mAh/g gravimetric and 830 mAh/cm^3^ volumetric capacities. One of the most common strategies to increase those values is to incorporate Si into the anode material. Silicon has 10-fold higher theoretical capacities, but its extreme volume expansion (∼300%) limits its use. Use of SiO as anodic material has been the subject of intense research, as it has superior cycling performance due to its limited volume expansion at about 20%,^49^ while still having high theoretical capacity, ranging from 1700 to 2400 mAh/g.^50^

However, the processing of SiO into nanoparticles can be a complex process. While there are many routes to prepare them (e.g., ball milling, CVD, deposition from gas-phase laser ablation, etc.), they often require multistep, time-consuming, and energy-intensive products and then need to be incorporated into the final anode preparation process. Moreover, the processing of nanomaterials, as previously mentioned, requires increased care and safety procedures as well as specialized equipment.

Therefore, a single-step process in which SiO microparticles are directly converted to nanoparticles concurrently with the production of a porous graphitic matrix to receive a self-standing, binder-free anode has many benefits, and the electrochemical results of a half-cell produced in this way are presented in Figure 6.

**Figure 6:**
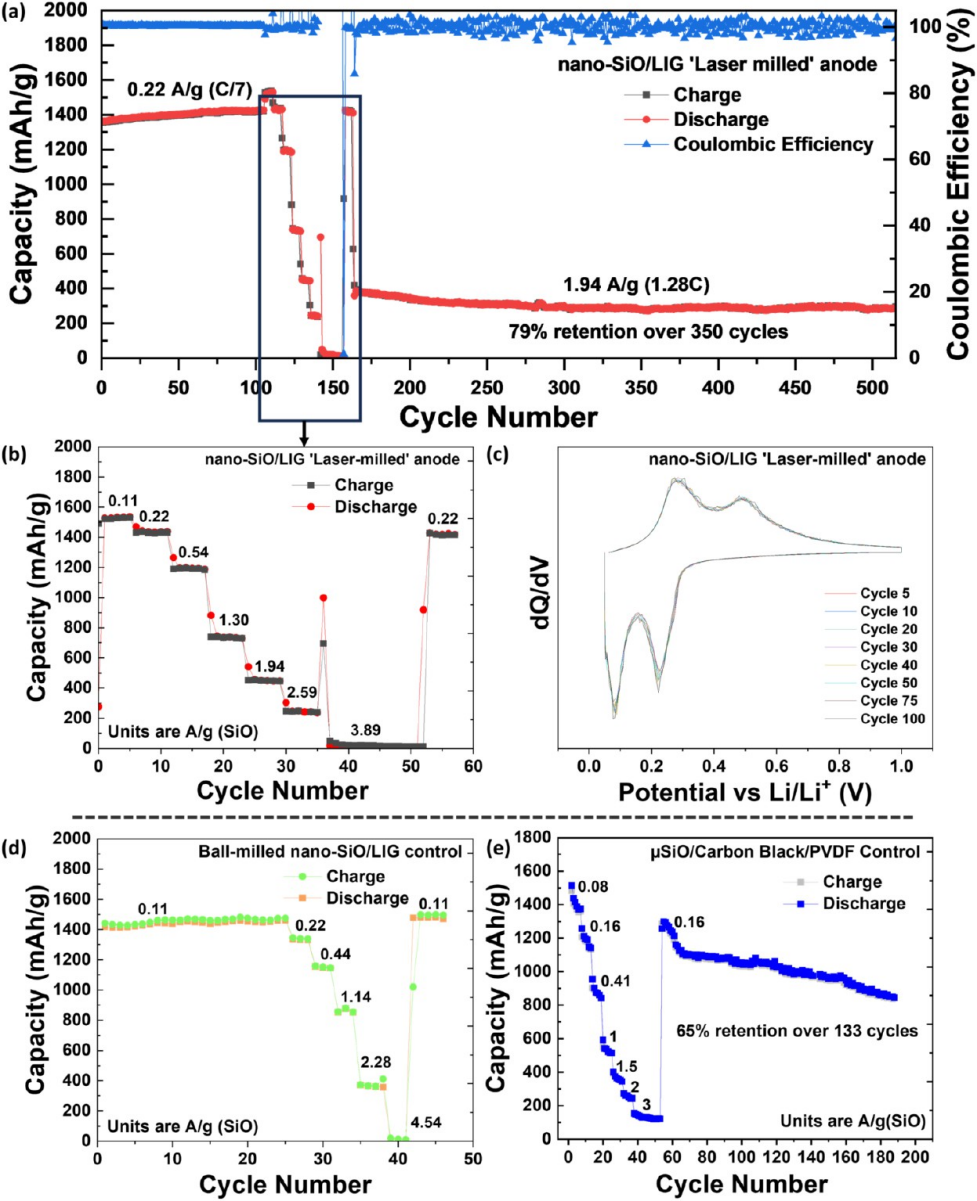
Half-cell evaluation of the SiO/LIG anode material: (a) Long-term cycling stability. (b) Rate performance analysis (current density in A/g (SiO)). (c) Differential capacity analysis demonstrating long-term stability. (d) Rate performance and cycling stability of a control anode composed of a mechanically ball-milled SiO/LIG composite at a comparable loading to the anode in (a). (e) Rate performance and cycling stability of a control anode using pristine SiO with standard binders and conductive additives at a comparable loading to the anode in (a).

Furthermore, the flexibility of the electrode is paramount for widespread application in next-generation electronics. Supporting Figure S6 displays the “laser-milled” SiO/LIG electrode on Cu foil, demonstrating flexibility and mechanical robustness. This structural integrity aligns with previously reported flexible, binder-free LIG-based composites for LIBs.^23,27^

Galvanostatic cycling of a microparticle-derived SiO/LIG anode at 0.22 A/g (SiO) (correlates to ∼C/7), in between 0.05 and 1 V vs Li/Li^+^, was conducted for 105 cycles, over 3 months of cycling, and is displayed in Figure 6a. Initial capacity was 1364 mAh/g and Coulombic efficiency at >99.99%, and remained stable over the duration of the experiment. The capacity slightly increased to 1430 mAh/g, which is attributed to a slow wetting process of deeper regions in the solid matrix of the anode material. Overall, the anode showed remarkable stability on par with state-of-the-art results, detailed in further paragraphs.

Following this test, the same cell was subjected to rate-capability analysis at various current densities (detailed in Figure 6b) and then cycled continuously at a high current density of 1.94 A/g (1.28C) for >350 more cycles, after C-rate analysis cycling was performed, showing a capacity retention over approximately 79% during the cycling at this rate (from cycle 160 in the graph). This further corroborates the validity of “laser-milling” as an applicable technique in many different use-cases, as the resulting composite undergoes minimal changes during operation (no loss of active material, etc.), while showing significant room for improvement in future research in fast charge/discharge performance for batteries produced in these techniques.

Further insight into the electrical characteristics of the “laser-milled” SiO/LIG anode is provided by Supporting Figure S7, which shows the electrochemical impedance spectroscopy (EIS) results. The Nyquist plot reveals a low charge-transfer resistance (*R*_ct_) of approximately 95 Ω, indicating the formation of a highly conductive carbon framework that facilitates efficient electron transport throughout the composite. This low interfacial resistance further supports the strong electrical connectivity within the LIG matrix and the stability of the SiO nanoparticles during cycling.

As the use of microparticles, SiO among them, is the subject of intense research, several other researchers have produced promising results. Li et al.^51^ have demonstrated a micro-SiO battery with an impressive gravimetric capacity of 1500 mAh/g, with degradation of >30% over 500 cycles at 0.5C. However, the composite production process requires multistepped reactions using multiple specialty polymers and binders (e.g., poly­(diallyldimethylammonium chloride)), adsorption, and washing steps, which reduce reaction yields and limit scale-up potential. Zhong et al.^52^ have reported an ultrahigh capacity anode based on graphene oxide and SiO, reaching a capacity equivalent to ∼1497.8 mAh/g, yet their process again suffers from high complexity: freeze-drying and several annealing steps in inert atmosphere. Bian et al.^53^ have recently shown a unique multielement anode (Mg and SiO), with an initial capacity of 1446.4 mAh/g, with ∼50% capacity fade over 200 cycles, with a relatively simple process that includes heating in inert atmosphere at 500–900 °C. The intense research in this field demonstrates the potential gains that can be made, especially when the simplicity of the “laser-milling” process is taken into consideration.

Further galvanostatic cycling at varying current densities was done to assess the rate performance of the SiO/LIG composite and is displayed in Figure 6b. This was achieved by increasing both charge and discharge rate every 5 cycles, starting from 0.11 A/g­(SiO) (C/14) to 3.89 A/g­(SiO) (2.5C). The capacity for cycling at 0.11 A/g (C/14) is ∼1520 mAh/g and changes to ∼1195 mAh/g when increasing the rate 5-fold to 0.54 A/g (C/3). This difference can be attributed to increased ion diffusion distances in the active material itself as well as concentration-polarization effects. Following cycling in fast charge/discharge rates, 5 more cycles at the standard 0.22 A/g rates show full recovery to the long-term cycling capacity at ∼1416 mAh/g.

Differential capacity analysis (DCA) (Figure 6c) further cements the high stability of the SiO/LIG as an anode material, showing peaks at 0.08 V and 0.24 V for lithiation and 0.29 V and 0.46 V for the delithiation reaction. This agrees with the literature and confirms that the lithiation and delithiation of SiO are the reversible reactions taking place.^54^ Furthermore, the lack of peak shifting or changes to the area under the peaks shows the stability of the anodic material. This underscores the stability of the SiO/LIG, showing that it does not undergo any meaningful phase change or loss of active material.

Figure 6d presents the performance of a control SiO anode that was mechanically ball-milled from a SiO micropowder into nanoparticles, followed by mixing with a phenolic resin precursor and subsequent laser ablation. This control electrode demonstrates cycling stability and rate capability comparable to those of the microparticle-derived laser-processed SiO/LIG anode shown in Figure 6a, under similar areal loadings and current densities. These results confirm that the electrochemical behavior of “laser-milled” SiO is on par with that of mechanically nanostructured SiO, validating the concept as an effective and scalable approach to achieve nanoscale performance starting from micropowder feedstock.

In contrast, Figure 6e shows the control electrode fabricated from pristine micro-SiO powder mixed with conventional conductive carbon black and a PVDF binder. Despite a similar initial capacity, this electrode exhibits rapid capacity fading and poor rate performance. The degradation arises from the severe volume expansion and pulverization of large SiO particles during cycling, leading to the loss of electrical contact, detachment from the current collector, and active material isolation. These effects result in pronounced capacity loss and highlight the limitations of pristine micro-SiO for stable long-term operation.

Taken together, Figure 6d,e clearly demonstrates the decisive impact of particle size on electrochemical performance. Nanoscale SiO, whether achieved through mechanical milling or laser processing, effectively mitigates the challenges of volume expansion, enabling improved structural integrity, cycling stability, and rate performance. The “laser-milling” process, however, offers the best of both worlds: it begins with inexpensive micropowder but produces a nanostructured, conductive SiO/LIG composite in a single, scalable step, combining manufacturing simplicity with the superior electrochemical properties of nanosized materials.

Post-mortem analysis of the SiO/LIG anode following several hundred cycles was conducted via SEM and EDS, as detailed in Supporting Figure S8. These results reveal several critical structural advantages. First, the porous nature of the matrix remains remarkably intact; the voids have not been ″clogged″ by excessive SEI formation, demonstrating that the LIG backbone is mechanically robust enough to buffer the characteristic volume expansion of silicon-based anodes without structural failure.

Furthermore, EDS mapping shows a highly homogeneous fluorine (F) content throughout the electrode, indicating the formation of a stable, thin SEI layer. This lack of ″infinite SEI growth″ explains the high electrochemical stability observed, directly addressing the primary degradation mechanism that typically plagues Silicon-based anodes. Consequently, the single-step conversion of SiO microparticles into a porous graphitic matrix provides a self-standing, binder-free solution with significant performance benefits.

To conclude the performance validation, a full cell analysis of the SiO/LIG laser-milled anode was conducted with a lithium iron phosphate (LFP) cathode. Even at high cycling rates (1C), the device demonstrated high capacity (>75 mAh/g) and stability, as detailed in Supporting Figure S9. These results further demonstrate the practicality for high-power applications and showcase the feasibility of this binder-free composite in integrated energy storage systems.

### Mechanistic Discussion

To elucidate the physical origins of the single-step dimensionality reduction observed in this study, we must first recap the structural transformations revealed by our results. We observed that microparticles of SiO, Si, and Mg, ranging from several micrometers to tens of micrometers, underwent a catastrophic transformation into uniform spherical nanoparticles embedded within the LIG matrix. Selected areas of the LIG surface even featured micrometer-sized craters surrounded by nanoparticle dispersions, as shown in Figure 1b. This behavior stands in stark contrast to that of the Al_2_O_3_ precursors, which, despite identical laser processing, exhibited only minor surface deformations without size reduction. This dichotomy implies a threshold-dependent mechanism governed by the thermal limits of the precursor materials relative to the extreme environment of LIG formation. Previous investigations into LIG synthesis have established that the localized absorption of the high-density photon flux generates temperatures commonly reported in the range of 2000–2500 K, accompanied by pressures on the order of gigapascals.^55,56^ Under such nonequilibrium conditions, materials are subjected to millisecond heating rates that far outpace conventional thermal diffusion.

We narrowed the potential mechanisms for this transformation down to three distinct physical pathways, comprising thermal stress-induced fracture, surface melting, and explosive boiling. The morphology of the resulting products allowed us to systematically rule out the first two options. Thermal stress fracture is driven by rapid expansion and crack propagation through the bulk particle, which would inevitably yield angular irregular fragments with a broad multimodal size distribution. This is fundamentally inconsistent with the high degree of sphericity and narrow size distribution observed in our results. Furthermore, as well-established in the fracture mechanics literature, there is no physical basis for brittle fracture to produce perfect spheres. Conversely, surface melting could theoretically account for the sphericity of the particles via surface tension forces. However, if surface melting were the dominant mechanism, we would expect to see many bulk microparticle remnants that remained solid or partially molten, surrounded by smaller satellite droplets stripped from the surface. This morphology is notably similar to what we observed with the alumina control sample in Figure 2e, where the bulk volume remained intact. This is distinct from prior studies on LIG-silicon composites, where irregular premade nanoparticles were simply spherized via melting without significant size reduction.^23^ Here, we observe both massive particle size reduction and spherization, indicating that bulk particle volume is involved in the transition.

To empirically validate this hypothesis, we performed a controlled experiment by irradiating a precursor blend with a very low concentration of SiO microparticles, as depicted in Figure 7a. This dilution maximized the interparticle distance, as shown in the inset of Figure 7a in optical microscopy imaging, allowing us to isolate single “explosion” events without overlap from neighboring interactions.

**Figure 7:**
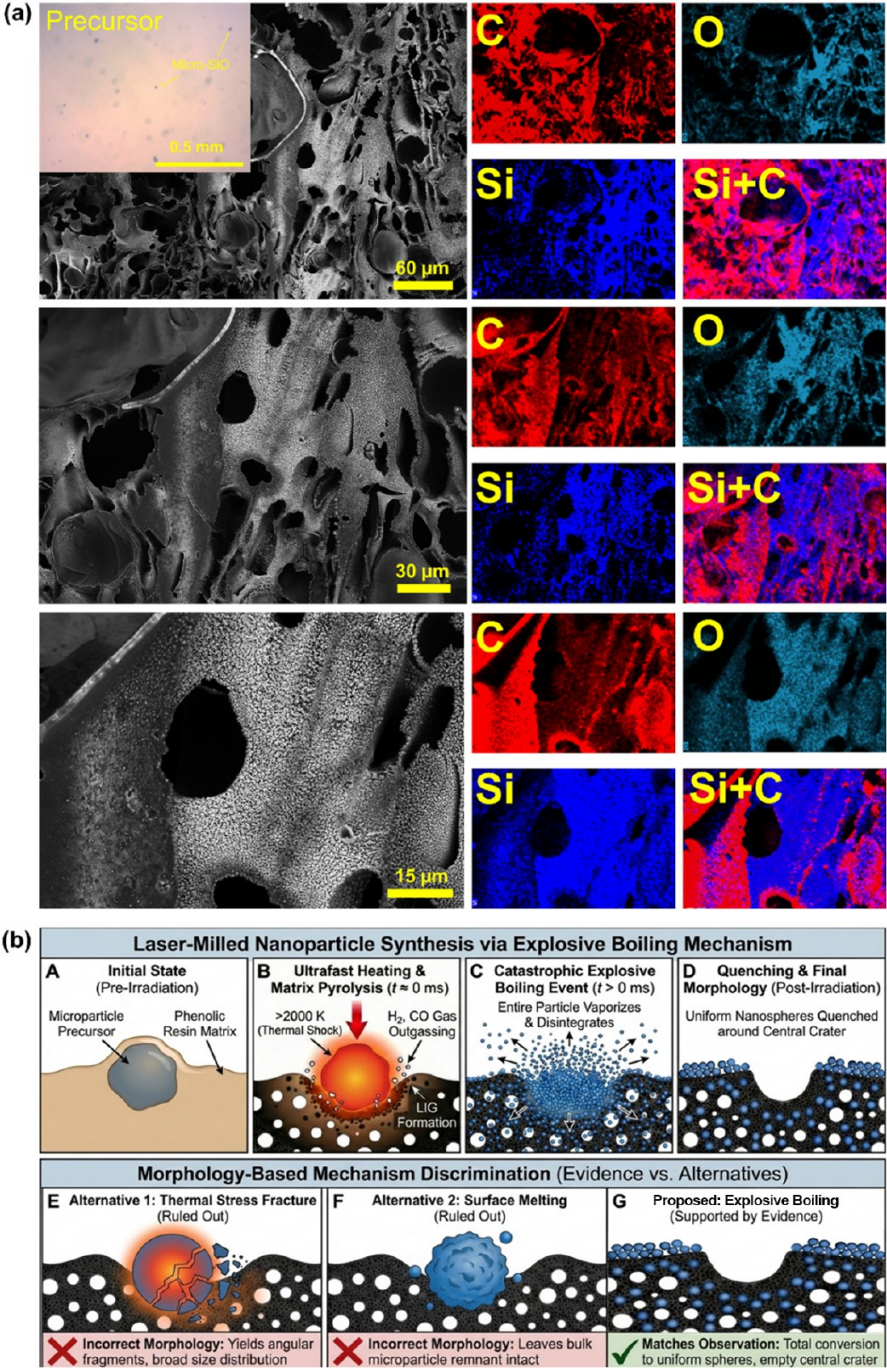
(a) SEM and EDS analysis showing the single-step transformation of isolated SiO microparticles into spherical nanoparticles embedded within the LIG matrix, showing a single “blast radius” of an isolated explosion. The inset shows optical microscopy of the dilute precursor blend. (b) Schematic illustration of the proposed explosive boiling mechanism distinguishing the volumetric disintegration pathway from thermal stress fracture and surface melting models.

The SEM and EDS maps reveal discrete micrometer-scale blast radii where a central cavity exposes the carbon support, surrounded by a “halo” of ejected silicon-based nanoparticles. The complete absence of a central residual microparticle within this “blast zone” provides direct visual evidence of the catastrophic nature of the event. Conversely, when this same isolation experiment was performed with alumina, as shown in Supporting Figure S10, we observed lone, intact microparticles sitting within the LIG matrix, confirming that without the explosive phase transition, the particle remains physically distinct. Figure 7b schematically summarizes the ruled out thermal cracking and surface melting mechanisms and the proposed explosive boiling mechanism, where the laser induces a rapid thermal shock that drives the microparticle into a superheated state, followed by volumetric disintegration and quenching within the forming LIG scaffold.

Consequently, we posit that explosive boiling, or phase explosion, is the most plausible mechanism to describe these observations.^44,47^ This phenomenon is critically dependent on the heating rate; under slow or equilibrium heating, the phase transition would proceed via heterogeneous nucleation at surface defects, allowing the material to undergo standard surface evaporation or gentle boiling. In our regime, however, ultrafast laser heating could drive the material into a superheated liquid state too rapidly for these equilibrium relaxation pathways to function. Once the material enters this superheated liquid phase, it becomes thermodynamically unstable, leading to homogeneous nucleation of vapor bubbles throughout the volume rather than just at the surface, and the internal pressure spikes rapidly. The subsequent volumetric expansion causes catastrophic fragmentation and violently tears the liquid apart into fine droplets. Concurrently, the significant outgassing generated by the resin decomposition further drives the dispersion of these droplets, which are subsequently quenched and immobilized upon the formation of the LIG scaffold.

This is justified kinetically by comparing the thermal relaxation time of the microparticles to the laser heating duration. Given the continuous laser spot diameter of approximately 50 μm and a scan speed of 4 mm/s, the effective dwell time is roughly 12.5 ms. For a silicon microparticle, the characteristic time for heat diffusion is on the order of microseconds (*t* ≈ *r* ^2^/α) which is significantly shorter than that of the millisecond-scale LIG formation process. This places the microparticles in a thermally thin regime, where the high thermal diffusivity allows heat to equilibrate rapidly throughout the volume, minimizing internal temperature gradients.

In this mechanism, the perfect sphericity of these resulting nanoparticles is thus a direct consequence of surface tension minimization acting on the liquid droplets during their brief flight time before quenching, a phenomenon well-described by the plateau-Rayleigh instability principles. This liquid-phase mediation explains why no angular fragments are observed and confirms that the material must have crossed the melting threshold prior to disintegration.

This hypothesis is further supported by the thermodynamic properties of the precursor materials. The resistance of alumina to this explosive process can be attributed to an energy barrier in its possible role as a thermal sink. Alumina possesses a high melting point (2072 °C) and an exceptionally high enthalpy of fusion (109 kJ/mol). In contrast, silicon melts at 1414 °C with an enthalpy of fusion of only 50 kJ/mol and magnesium melts at 650 °C with an even lower enthalpy of 8.48 kJ/mol. The laser energy absorbed by alumina is largely consumed in overcoming this massive enthalpic barrier to melting, effectively clamping the temperature near the melting point and preventing accumulation of the excess energy required for superheating. Conversely, Si and Mg overcome their melting barriers rapidly, possibly allowing the remaining laser fluence to drive the liquid melt into the explosive metastable regime.

## Conclusions

This study introduces a single-step top-down nanoparticle synthesis and composite integration method, achieved via simultaneous “laser-milling” of microparticle precursors and the formation of a laser-induced graphene (LIG) matrix. Such a strategy enables concurrent fabrication and integration of nanoparticles and porous graphitic carbon frameworks from microparticle and commercial polymer precursors. Size reduction is hypothesized to occur through synergistic effects of rapid localized heating, gas evolution, and explosive boiling, yielding stable nanoparticle dispersions embedded directly in a conductive LIG scaffold. The scalability and versatility of “laser milling” are evident in its ability to form various active nanoparticles within a 3D conductive matrix while minimizing process complexity, material waste, and reliance on binders or additives.

The resulting microparticle-derived Mg/LIG, SiO/LIG, and Si/LIG nanocomposites exhibit uniformly dispersed nanoparticles and high conductivity, highlighting the advantages of this single-step in situ process. These monolithic, self-supporting structures feature intimate interfacial interactions between the nanoparticles and the LIG matrix, which provide structural integrity alongside enhanced electrochemical performance. Thus, this architecture yields a composite that offers the processing ease of microparticles and the performance of nanoparticles, all anchored within a structural graphitic support.

This study demonstrates the practical and functional benefits of the proposed approach through electrochemical testing of a microparticle-derived SiO/LIG anode for lithium-ion batteries, which exhibited significantly improved conductivity and cycling stability compared with a commercial microparticle counterpart. Notably, the anode also showed performance comparable to an analogous SiO/LIG nanocomposite synthesized from premade nanoparticles. Such parity indicates that “laser-milling” effectively reduces microparticle precursors into high-quality nanoparticles in situ, achieving the same electrochemical benefits as traditional nanoprecursors without requiring separate, complex synthesis steps. The LIG scaffold facilitates efficient charge transport and accommodates structural changes during cycling, enhancing the overall resilience of silicon-based anodes. Such results validate “laser-milling” as a broadly applicable strategy for energy storage and beyond, as evidenced by successful nanoparticle formation from Si, SiO, and Mg precursors.

Looking forward, further optimization of laser parameters and exploration of diverse precursor systems could extend the applicability of this technique. Its compatibility with scalable manufacturing approaches, such as roll-to-roll processing, highlights its promise as a rapid, single-step approach for next-generation nanocomposites tailored to the demands of catalysis, sensing, and energy technologies.

## Supplementary Material



## Notes

Spectroscopy analyses, microscopy images, additional measurements, and electrochemical characterization (PDF)
