# Supplementary material for: A Versatile Single-Step Micro- to Nanoparticles Laser Graphitization-Driven Conversion Route for Graphene-Embedded Nanoparticle Composites
Source: ACS Appl Mater Interfaces. 2026 Mar 11;18(11):16899–914. doi: 10.1021/acsami.6c00908 (PMC13022816; doi:10.1021/acsami.6c00908)
Supplement: Supplementary file 1 [file am6c00908_si_001.pdf]

Supporting Information

A Versatile Single-Step Micro-to-Nanoparticles Laser Graphitization-Driven Conversion Route for Graphene-Embedded Nanoparticle Composites

Assaf Eran [1#], Gil Daffan [1#] and Fernando Patolsky [1,2,3*]

1. Department of Materials Science and Engineering, Faculty of Engineering, Tel Aviv University, Tel Aviv 69978, Israel.

2. School of Chemistry, Faculty of Exact Sciences, Tel Aviv University, Tel Aviv 69978, Israel.

3. Tel Aviv University Center for Nanoscience and Nanotechnology, Tel Aviv University, Tel Aviv 69978, Israel.

# These authors contributed equally to this work.

*Corresponding author. Email: fernando@tauex.tau.ac.il

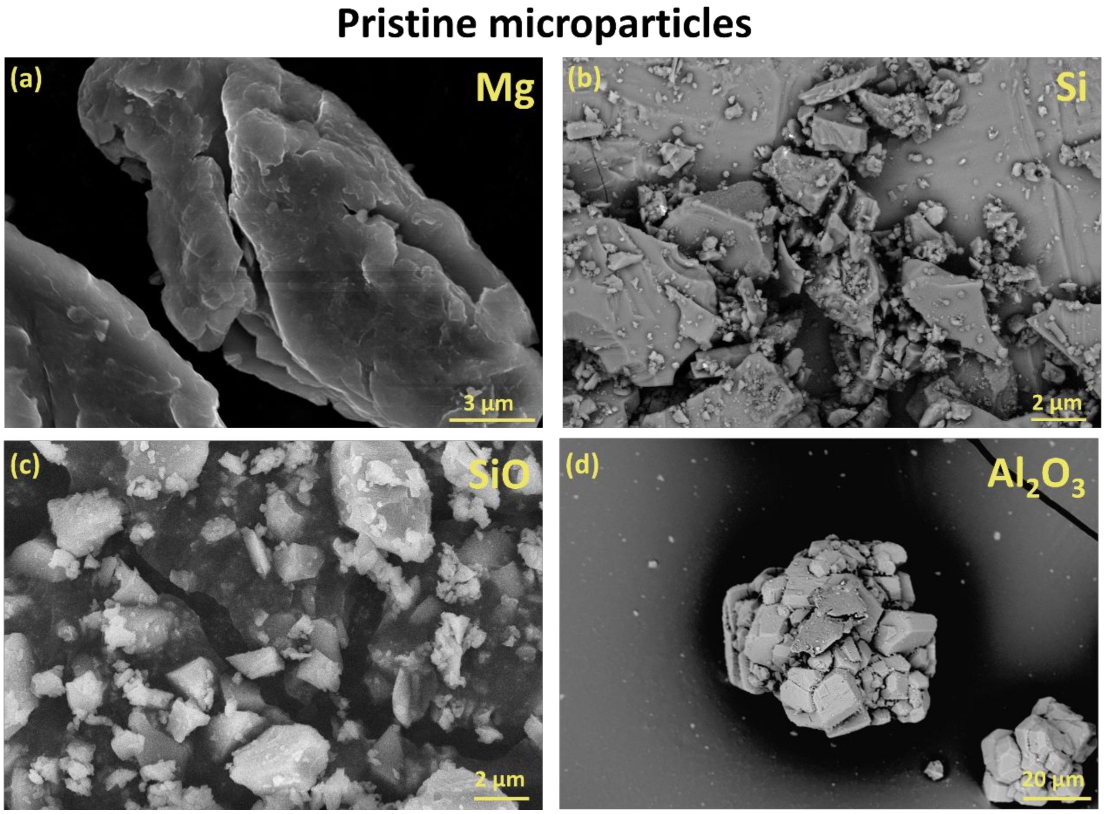

Figure S1. SEM imaging of the pristine microparticles as purchased, providing a baseline for comparison before any laser processing. The images show the initial morphology, size distribution, and surface characteristics of the precursor powders. These micrographs confirm that the starting

materials consist of microparticles of varying sizes, which is essential for evaluating the efficacy of the subsequent laser milling process and the resulting dimensionality reduction to the nanoscale.

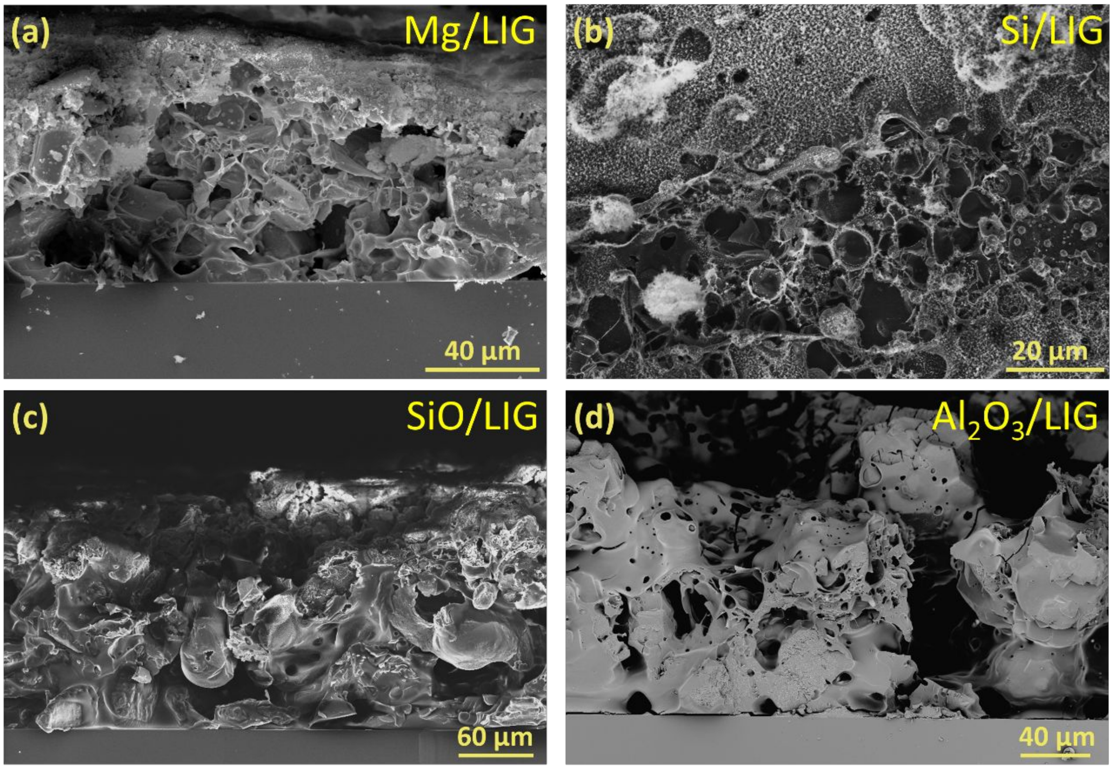

Figure S2. SEM cross-sectional imaging of the 'laser-milled' composite structures. (a) Mg/LIG (b) Si/LIG (c) SiO/LIG and (d) Al_2O_3/LIG. The micrographs indicate that the porous morphology characteristic of LIG is maintained throughout the electrode thickness. Notably, for the Mg, Si, and SiO composites, there is an absence of discernible microparticles within the cross-sectional plane, suggesting a high degree of volumetric conversion via the laser milling process. The preservation of this porous network throughout the depth of the material is critical for facilitating electrolyte transport and providing the structural voids necessary to accommodate volumetric changes during operation. These lasing parameters for full irradiation depth transformation were optimized with the same laser system in previous studies [1–3].

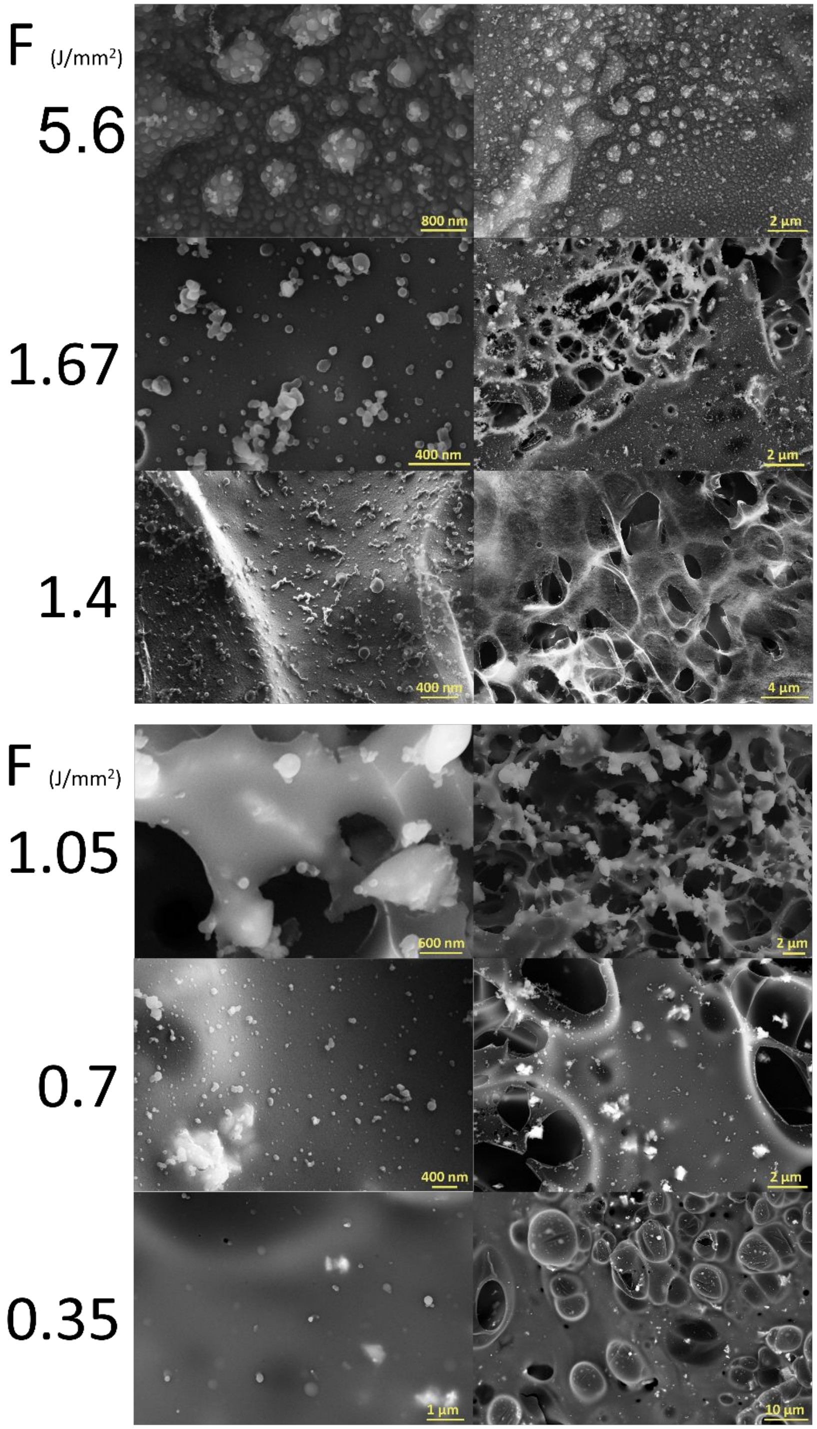

Figure S3. SEM imaging of the surface of SiO/LIG composites irradiated under different laser fluences. Optimized laser fluence range was observed in previous studies[1,3] .Particle size remains consistent within this optimized growth window, with a lower particle density threshold observed at 0.35 J/mm^2.

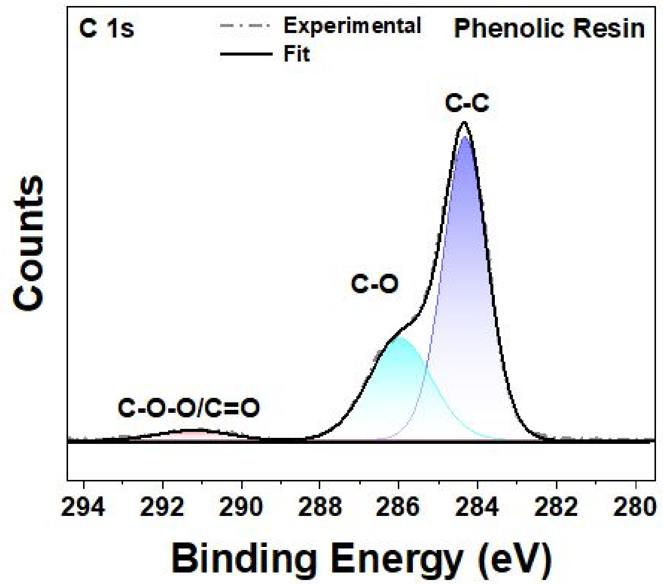

Figure S4. XPS C 1s spectra of the phenol-formaldehyde resin (PR) LIG precursor. In comparison to the C 1s XPS spectra of LIG in Figure 5 of the main text, the notable disappearance of C–O and C=O bonds after the lasing process highlights the extent of graphitization, which proceeded without being hindered by nanoparticle formation.

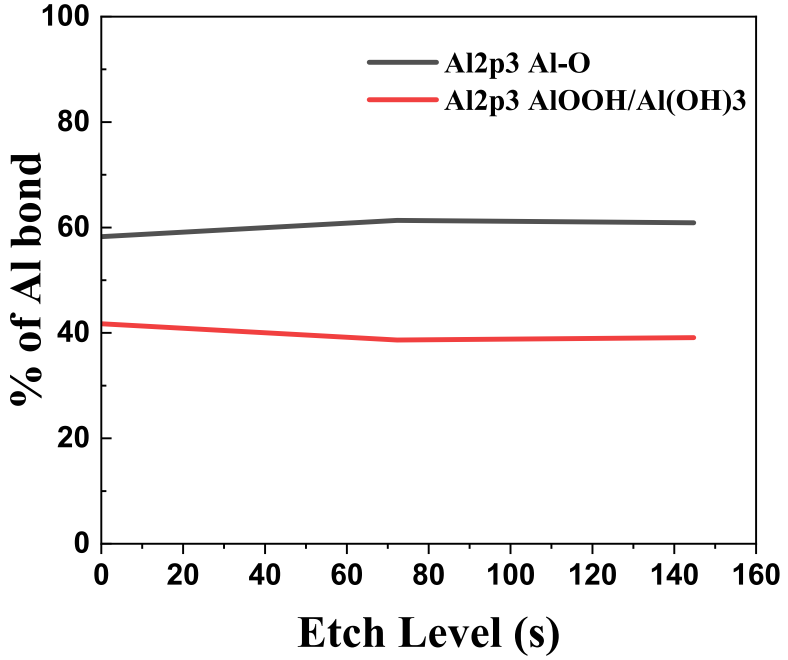

Figure S5. XPS depth profile of the Al_2O_3/LIG sample, showing increased surface hydroxylation. This indicates slight surface reactivity of the alumina during the laser process, even though no nanoparticles were formed due to the thermal properties and high stability of the Al_2O_3 microparticles. The depth profile reveals that while the bulk of the microparticle remains unchanged, the intense photothermal environment induces localized chemical modifications at the interface.

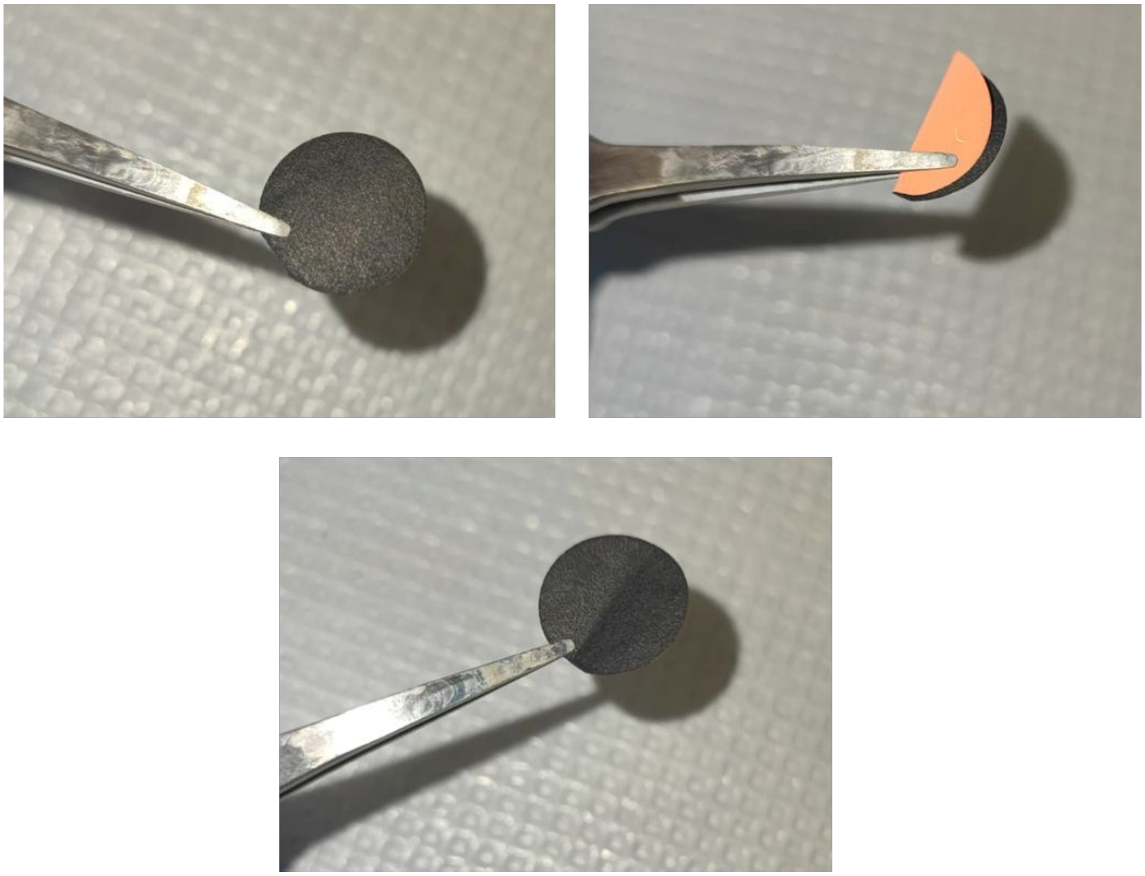

Figure S6. Flexible bending of the SiO/LIG electrode coated on battery-grade copper foil. Visual analysis shows no delamination or cracking before and after repeated bending. This mechanical integrity is critical for battery applications, as it ensures continuous electrical contact and active material retention during physical deformation. These results are consistent with previous reports on flexible, self-standing, binder-free electrodes made with LIG [1–3], confirming the robust adhesion and structural stability of the composite framework.

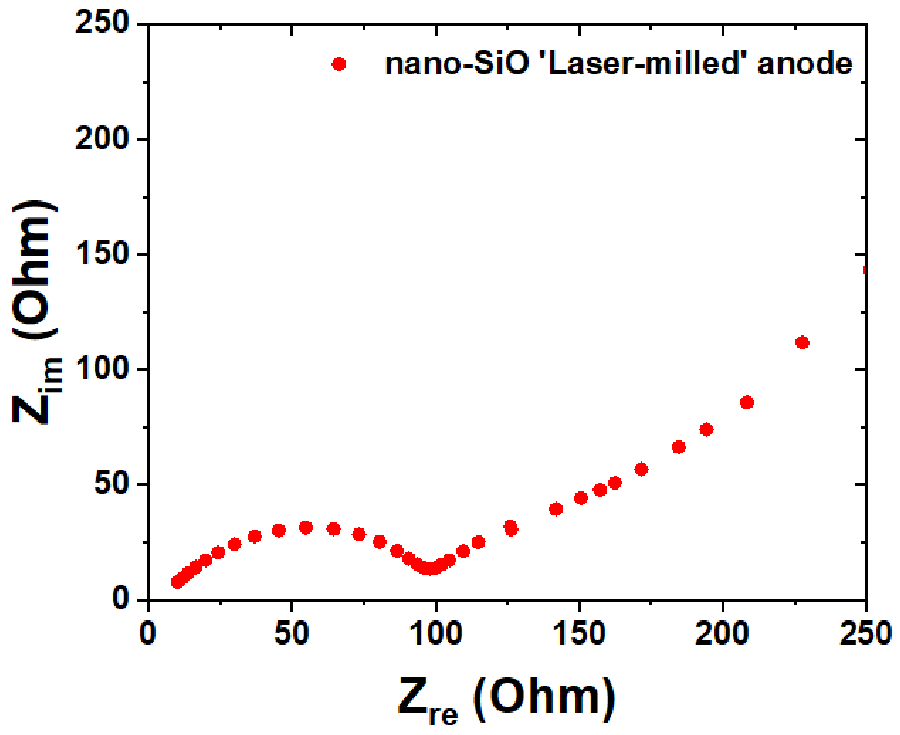

Figure S7. Electrochemical impedance spectroscopy (EIS) of the 'laser-milled' SiO/LIG anode. The Nyquist plot shows a small semicircle in the high-to-medium frequency region corresponding to a charge-transfer resistance (R_{ct}) of approximately 95 Ω. This low resistance is attributed to the conductive graphitic matrix backbone, which provides an efficient path for electron transport and is critical for high-rate anode performance. The stable LIG framework ensures continuous electrical contact with the SiO nanoparticles throughout the electrochemical cycling process.

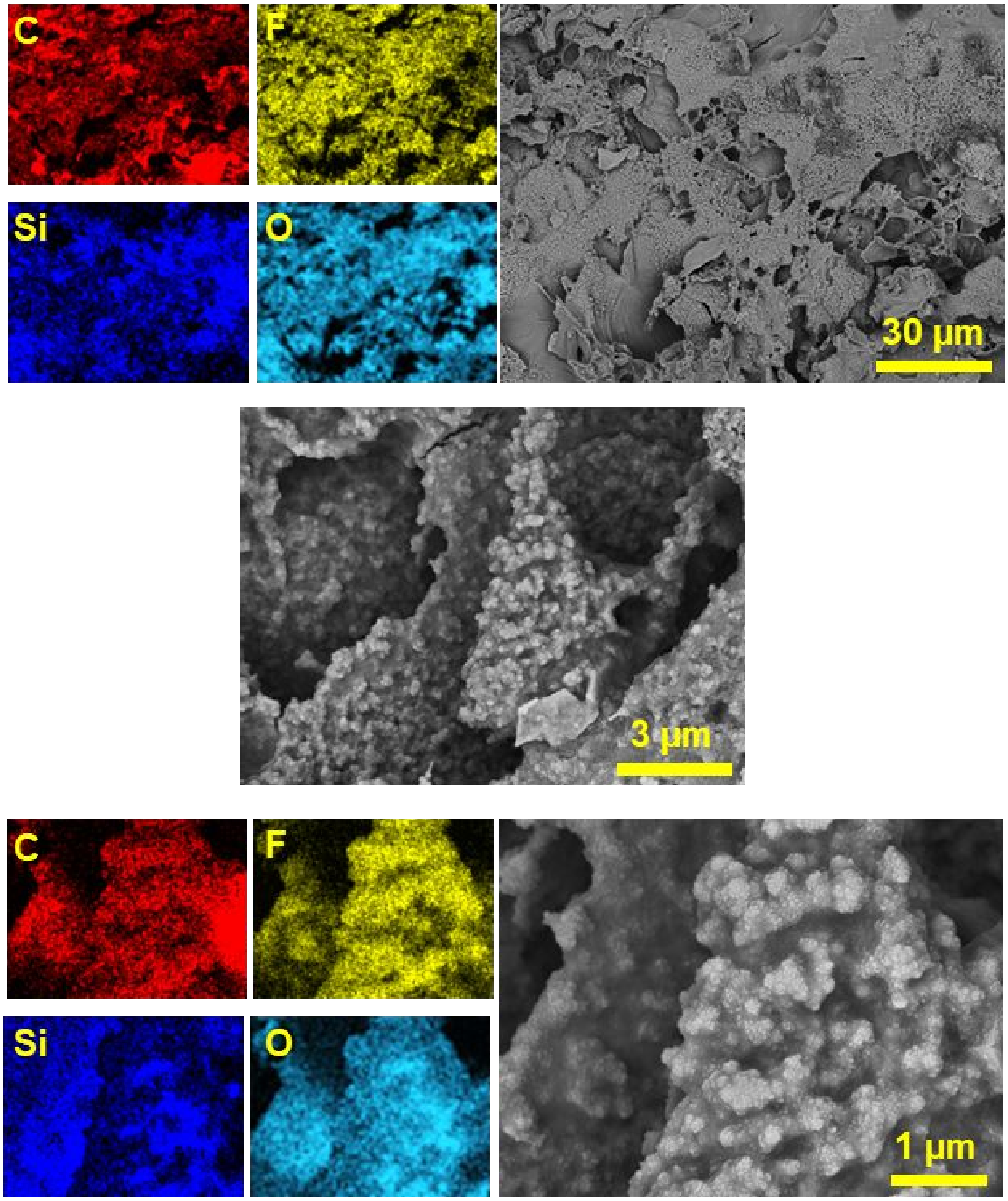

Fig. S8. Post-mortem SEM and EDS analysis of the laser-milled SiO/LIG anode derived from SiO microparticle precursors after hundreds of cycles in a lithium-ion battery half-cell. Analysis shows the porous structure remained intact, indicating that Si volume expansion was effectively suppressed and buffered by the mechanically resilient LIG scaffold. The SEM images confirm a homogenous distribution of particles is maintained, while EDS fluorine (F) elemental mapping reveals a thin, conformal SEI layer. This stable interface and robust framework demonstrate the mechanical resilience of the composite during long-term cycling.

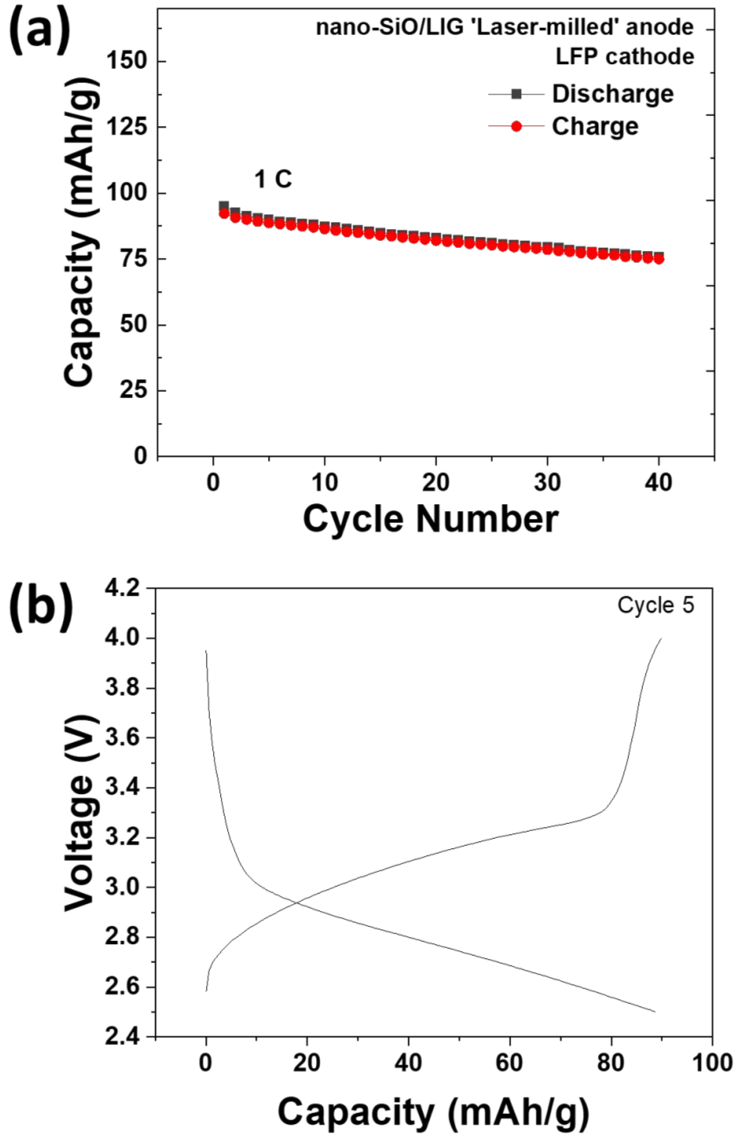

Figure S9. Full-cell electrochemical performance of the LFP || 'Laser-milled' SiO/LIG system. (a) Galvanostatic cycling performance and capacity retention evaluated at a 1C discharge/charge rate. (b) Representative galvanostatic voltage profiles displaying the charge/discharge curve.

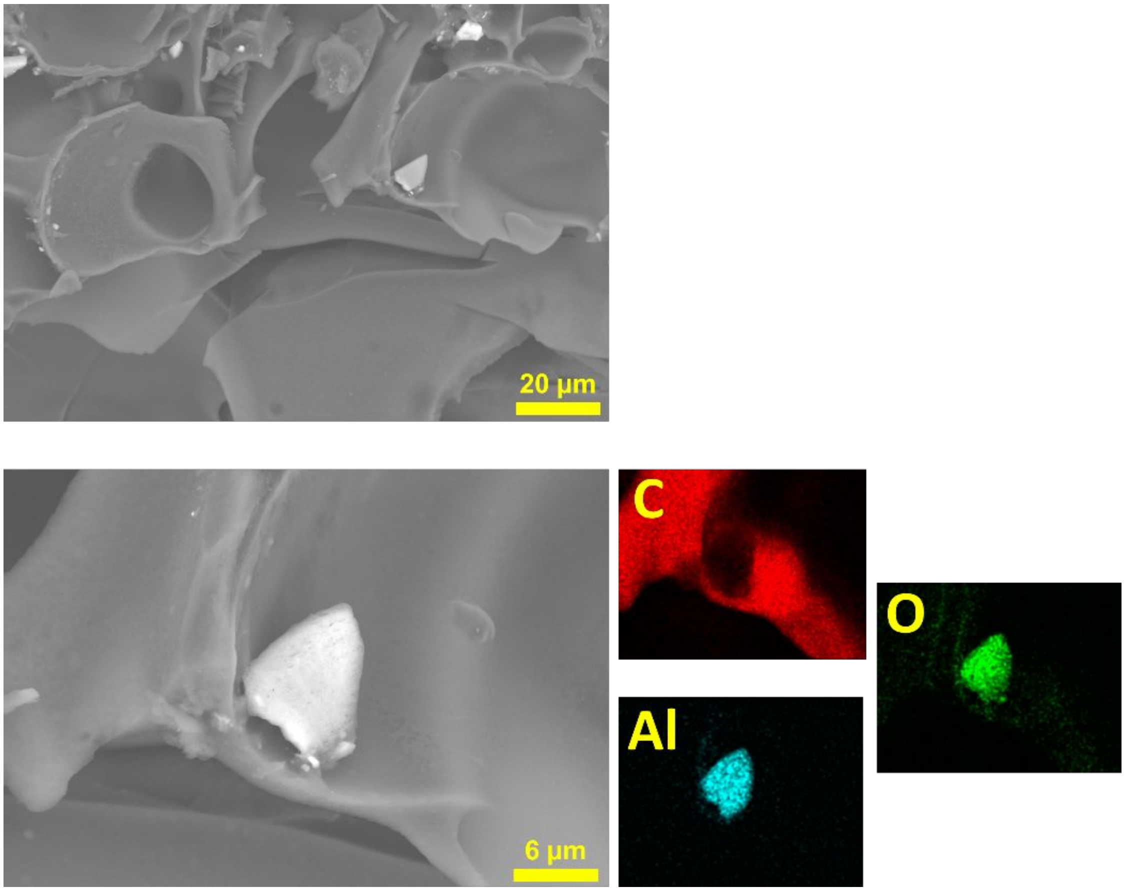

Figure S10. SEM and EDS imaging of an Al_2O_3/LIG composite prepared from a dilute blend of alumina microparticles in phenolic resin. The analysis confirms that no nanoparticle formation occurred and original microparticles remain intact, establishing a thermal threshold for the process. This is in stark contrast to the analogous SiO based experiment in Fig. 7a, where significant particle size reduction was observed. These results indicate that the temperatures reached during laser induced graphitization are insufficient to drive the dimensionality reduction for Al_2O_3, highlighting the material specific limitations of the 'laser milling' mechanism.

References

(1) Daffan, G.; Kothuru, A.; Eran, A.; Patolsky, F.; Daffan, G.; Eran, A.; Patolsky, F.; Kothuru, A. In-Situ Laser Synthesis of Molecularly Dispersed and Covalently Bound Phosphorus-Graphene Adducts as Self-Standing 3D Anodes for High-Performance Fast-Charging Lithium-Ion Batteries. *Adv. Energy Mater.* **2024**, 2401832. https://doi.org/10.1002/AENM.202401832.

(2) Kothuru, A.; Daffan, G.; Cohen, A.; Patolsky, F. Monolithic 'Sulfo-Graphene' Cathodes: Towards Ultrastable High-Performance Lithium–Sulfur Batteries. *Carbon N. Y.* **2026**, *248*, 121177. https://doi.org/10.1016/J.CARBON.2025.121177.

(3) Kothuru, A.; Cohen, A.; Daffan, G.; Juhl, Y.; Patolsky, F. Pioneering the Direct Large-Scale Laser Printing of Flexible "Graphenic Silicon" Self-Standing Thin Films as Ultrahigh-Performance Lithium-Ion Battery Anodes. *Carbon Energy* **2024**, e507. https://doi.org/10.1002/CEY2.507.
